# Identification of potential biomarkers for lung adenocarcinoma: a study based on bioinformatics analysis combined with validation experiments

**DOI:** 10.3389/fonc.2024.1425895

**Published:** 2024-09-19

**Authors:** Chuchu Zhang, Ying Liu, Yingdong Lu, Zehui Chen, Yi Liu, Qiyuan Mao, Shengchuan Bao, Ge Zhang, Ying Zhang, Hongsheng Lin, Haiyan Li

**Affiliations:** ^1^ Institute of Information on Traditional Chinese Medicine, Chinese Academy of Chinese Medical Sciences, Beijing, China; ^2^ Institute of Chinese Materia Medica, China Academy of Chinese Medical Sciences, Beijing, China; ^3^ Guang’anmen Hospital, Chinese Academy of Chinese Medical Sciences, Beijing, China; ^4^ College of Basic Medicine, Shaanxi University of Chinese Medicine, Xianyang, China; ^5^ Department of Cardiology, The First Affiliated Hospital of Zhengzhou University, Zhengzhou, China; ^6^ Key Laboratory of Cardiac Injury and Repair of Henan Province, Zhengzhou, China

**Keywords:** lung adenocarcinoma, TGFBR2, Mendelian randomization, machine learning, immune infiltration

## Abstract

**Background:**

The prognosis for lung adenocarcinoma (LUAD) remains dismal, with a 5-year survival rate of <20%. Therefore, the purpose of this study was to identify potentially reliable biomarkers in LUAD by machine learning combination with Mendelian randomization (MR).

**Methods:**

TCGA-LUAD, GSE40791, and GSE31210 were employed this study. Key module differential genes were identified through differentially expressed analysis and weighted gene co-expression network analysis (WGCNA). Furthermore, candidate biomarkers were derived from protein–protein interaction network (PPI) and machine learning. Ultimately, biomarkers were confirmed using MR analysis. In addition, immunohistochemistry was used to detect the expression levels of genes that have a causal relationship to LUAD in the LUAD group and the control group. Cell experiments were conducted to validate the effect of screening genes on proliferation, migration, and apoptosis of LUAD cells. The correlation between the screened genes and immune infiltration was determined by CIBERSORT algorithm. In the end, the gene-related drugs were predicted through the Drug–Gene Interaction database.

**Results:**

In total, 401 key module differential genes were obtained by intersecting of 5,702 differentially expressed genes (DEGs) and 406 key module genes. Thereafter, GIMAP6, CAV1, PECAM1, and TGFBR2 were identified. Among them, only TGFBR2 had a significant causal relationship with LUAD (p=0.04, b=−0.06), and it is a protective factor for LUAD. Subsequently, sensitivity analyses showed that there were no heterogeneity and horizontal pleiotropy in the univariate MR results, and the results were not overly sensitive to individual SNP loci, further validating the reliability of univariate Mendelian randomization (UVMR) results. However, no causal relationship was found between them by reverse MR analysis. Meanwhile, TGFBR2 expression was decreased in LUAD group through immunohistochemistry. TGFBR2 can inhibit proliferation and migration of lung adenocarcinoma cell line A549 and promote apoptosis of A549 cells. Immune infiltration analysis suggested a potential link between TGFBR2 expression and immune infiltration. Finally, Irinotecan and Hesperetin were predicted through DGIDB database.

**Conclusion:**

In this study, TGFBR2 was identified as a biomarker of LUAD, which provided a new idea for the treatment strategy of LUAD and may aid in the development of personalized immunotherapy strategies.

## 1 Introduction

Lung adenocarcinoma (LUAD), which can be surgically resected at an early stage, has become one of most common pathological subtypes of non-small-cell lung cancer (NSCLC) (1). Unfortunately, LUAD is usually diagnosed at an advanced stage since its rapid progression. In clinical practice, only a minority of LUAD patients respond to chemo- or radiotherapy effectively, whereas a majority of them with metastasis of LUAD cells at advanced stage have high resistance to these therapies, thereby presenting a poor prognosis and high mortality at this stage, with a 5-year survival rate of <20% (2, 3). Thus, it is crucial to conduct early detection, diagnosis, and treatment in the clinical management of LUAD. Compared with other pathological subtypes of lung cancer, the occurrence of LUAD, as a peripheral type, is proven to be involved in multiple driver genes (4). Hence, screening and identification of LUAD signature genes play significant roles for these patients in early detection, personalized treatment, and enhancement of prognosis.

In recent years, bioinformatics methods have undergone significant advancement, and a wealth of disease databases have become available and improved, which bring new prospects and convenience for excavation of marker genes and further provide substantial value and basis in the field of diagnosis and treatment of diseases. With the boom of artificial intelligence, machine learning algorithms have become one of the frequently utilized technologies in bioinformatics study, including support vector machine (SVM), k-nearest neighbor (KNN), random forest (RF), naive Bayes (NB), convolutional neural networks (CNN), and autoencoder (AE), and these computational approaches have been extensively applied to identify disease markers, construct disease models, screen drugs, and support other medical research (5–8). Mendelian randomization (MR) refers to an analysis method for inferring causality based on genetic variation, mainly investigating the impact of naturally occurring random gene allocation of phenotypes on outcomes of diseases (9). Since these genes are allocated randomly, MR is much less susceptible to issues of causation and confounding bias than traditional tools. Thus, it enhances the reliability of causal inferences. At present, MR, as a powerful tool in bioinformatics mining, has been successfully applied across variety medical fields, notably contributing to the identification of targets for disease prevention and treatment (10).

**Figure 1:**
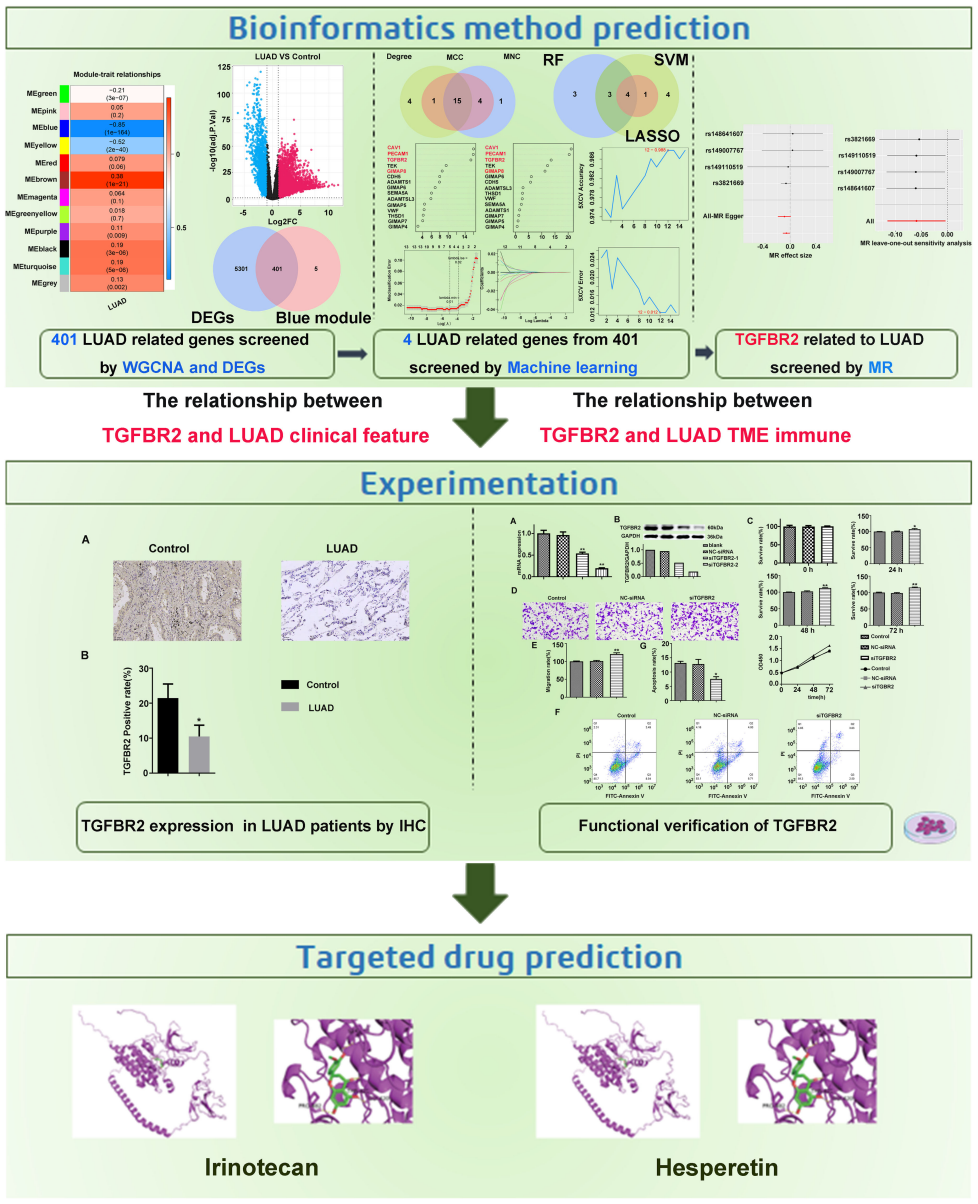
Design flowchart of research.

In our study, machine learning algorithms in conjunction with MR analysis were employed to more accurately identify the key genes that have causal relationships with LUAD. Subsequently, the effects of these ultimately screened genes were validated through their impacts on clinical tissue samples and lung adenocarcinoma cell lines. Meanwhile, acknowledging the crucial role of immune infiltration in tumors, the relationship between the screened genes and immune infiltration was analyzed. In addition, the potential drugs that interference with these targets via molecular docking were identified, with the expectation that our in-depth study on gene screening for LUAD provides a theoretical foundation and valuable insights for the early diagnosis and prevention of the disease. **Figure 1** depicts our research approach.

## 2 Materials and methods

### 2.1 Data source

In this study, TCGA-LUAD, which contained 515 LUAD and 59 control samples, was downloaded from the UCSC database for use as the training set. GSE40791 (11) and GSE31210 were obtained from the Gene Expression Omnibus (GEO) database (https://www.ncbi.nlm,nih.gov/geo) (12) for use as the validation set; GSE31210 included 226 LUAD and 20 control samples, while GSE40791 includes 94 LUAD lung tissue samples and 100 normal control lung tissue samples. In addition, the relevant GWASID datasets were downloaded from Integrative Epidemiology Unit (IEU) Open genome-wide association studies (GWAS) (https://gwas.mrcieu.ac.uk/). The LUAD-related dataset (ieu-a-984) consisted 65,864 samples (11,245 LUAD vs. 54,619 control samples) and included 10,345,176 single nucleotide polymorphisms (SNPs).

### 2.2 Weighted gene co-expression network analysis

The co-expression network was constructed using WGCNA package (13) to identify the gene modules with strong association with LUAD. Initially, the presence of any outliers among the samples was checked; subsequently, a soft threshold was determined to construct an adjacency matrix, which was then clustered to identify the central module. The module with the strongest positive correlation with LUAD genes was selected for further analysis, as determined by calculating the Pearson correlation coefficients. Afterwards, gene significance (GS) and module membership (MM) were calculated for each gene within the central module. Eventually, genes with MM >0.6 and GS >0.6 were considered to be candidate LUAD-associated genes. Lastly, functional enrichment analysis of the genes in the module was performed using the online Metascape database (https://metascape.org/gp/index.html#/main/step1), and the enrichment results were visualized as a bar chart.

### 2.3 Screening of candidate genes

In TCGA-LUAD, differentially expressed genes (DEGs) between LUAD and control samples were identified using “edgeR” (14) in R package, which adjust. P < 0.05 and |log2FC| > 1 (15). For the key module genes identified previously, thresholds of MM > 0.6 and GS > 0.6 were also utilized as part of the differential screening condition. Afterwards, DEGs and key module genes intersected the key module differential genes. The associated biological pathways and functions were then explored through enrichment analysis. Subsequently, a protein–protein interaction network (PPI) was constructed for key module differential genes. Then, the MCC, MNC, and degree algorithms were applied to select TOP20 genes, respectively, Eventually, the candidate hub genes were identified by finding the intersection of these selections.

### 2.4 Machine learning

Various machine learning techniques were employed to accurately screen candidate hub genes, specifically including random forest (RF), least absolute shrinkage and selection operator (LASSO), and support vector machine recursive feature elimination (SVM-RFE). Logistic regression, a widely used linear model for binary classification problems, outputs predictive probabilities and helps to assess the importance of features. For the LASSO model, the binary classification labels (LUAD/health) were converted into numerical labels (e.g., 1 for LUAD, 0 for health) to enable the model’s training and prediction. SVM is a powerful classification algorithm capable of handling non-linear data effectively, with generalization capabilities. RF is an ensemble method based on decision trees, capable of handling high dimensional data with strong robustness and predictive ability.

Primarily, random forest analysis was conducted using R-package “randomForest” (16) with default parameters, and the candidate genes were ranked based on their importance. Using the mean decrease gini algorithm, the top 10 genes were selected as key candidate hub genes. Second, LASSO regression analysis was performed on the expression of candidate hub genes using R-package “glmnet” (17), taking into account the clinical information of the samples. Genes that were more critical to the disease were penalized less, whereas genes that were less relevant to the disease were penalized more, potentially reducing their coefficients to 0, and a fivefold cross-verification was then performed. Ultimately, the SVM-RFE method was applied to determine the importance and rank of each gene. Fivefold cross-validation was set, and the error rate and accuracy of each iteration combination were obtained. The lowest error rate was selected as the best combination, and the corresponding gene was selected as the candidate key gene. Binary categorical variables, where disease is represented by 1 and control by 0, were used by machine learning algorithms to distinguish between disease and control statuses. The candidate biomarkers were obtained by finding the intersection of genes selected by the three machine learning algorithms.

### 2.5 Expression validation and Kaplan*–*Meier analysis of candidate biomarkers

The expression of candidate biomarkers was analyzed using R package “ggplot2” (18) in TCGA-LUAD, and this analysis was subsequently validated in GSE31210 and GSE40791. Furthermore, to assess the predictive power of candidate biomarkers, ROC curves of LUAD and normal samples were constructed in TCGA-LUAD, GSE40791, and GSE31210 using R packet “pROC” (19). A higher AUC value indicates a more accurate prediction. Subsequently, in TCGA-LUAD, patients were divided into high and low expression groups based on the optimal threshold of expression levels of the candidate biomarkers’ expression levels, and the survival differences between these groups were analyzed using K-M survival analysis.

### 2.6 Analysis of the causal relationship between exposure factors and outcome through univariate Mendelian randomization analysis

To identify instrumental variables (SNPs) reasonably associated with exposure factors, the extracting instruments function of the R package TwoSampleMR (version 0.5.6) (20) was utilized, with a threshold of p < 5×10^−8^ for significance. Instrumental variables showing linkage disequilibrium (LD) were excluded using the “clump” function with the argument “clump = TRUE.” Based on the outcome GWAS data and instrumental variables screened in the previous step, we then excluded instrumental variables that showed a significant association with the outcome.

In this study, “TwoSampleMR” R package was employed to conduct two-sample MR analysis between exposures and outcome. The five common MR methods were applied to features with multiple instrumental variables (IVs): MR-Egger regression (21), inverse-variance weighted (IVW) method (22), the weighted median test (23), the weighted mode test (24), and the simple mode test (25). The IVW method served as the primary approach for investigating. Subsequently, scatter plots were used to determine the correlation between exposure factors and LUAD. Then, the correlation between them was determined by the scatter plot. Forest was generated to assess to the efficacy of SNP site for predicting candidate biomarkers for LUAD diagnosis. Moreover, funnel plots were applied to determine whether the SNPs loci was randomized. Meanwhile, the study evaluated the heterogeneity, pleiotropy, and sensitivity of univariate MR analysis results. Lastly, to rule out the potential for reverse causality, reverse MR analysis was performed on key genes that were causally related to LUAD.

### 2.7 Analysis of the correlation between TGFBR2 gene and clinical features of LUAD

TCGA-LUAD data were used to explore the relationship between the expression of the TGFBR2 gene and various clinical factors such as age, gender, clinical staging, and tumor classification in LUAD.

### 2.8 Immune cell infiltration analysis

The Estimate software package was employed to analyze immune infiltration parameters in groups characterized by high and low TGFBR2 expression, specifically assessing matrix score, immune score, and composite score. The CIBERSORT (26) deconvolution algorithm was used to examine and depict the immune cell infiltration in these groups. A p < 0.05 denotes a significant difference in immune cell infiltration between the groups, which was further analyzed for Pearson correlation with TGFBR2 expression.

### 2.9 Immunohistochemical analysis

In order to examine the expression level of the gene found to have a causal relationship with LUAD, the expression of this gene was analyzed by immunohistochemistry (IHC). We obtained a total of five lung adenocarcinoma tissues and three normal lung tissues from the Pathology Department at Guang’anmen Hospital, China Academy of Chinese Medical Science. Suitable tissue samples were then prepared for paraffin embedding and section. First, the tissues were washed with phosphate-buffered saline (PBS) and then immersed in 4% paraformaldehyde for 48 h. Next, they were dehydrated through sequential immersion in ethanol of increasing concentrations. Following dehydration, the tissues were cleared in xylene before being infiltrated with paraffin. Finally, the tissues were placed in a mold containing paraffin, and the paraffin was removed after cooling and sliced for paraffin removal. In summary, the sections underwent deparaffinization and were then rehydrated before being incubated with primary antibodies (1:100 dilution, Proteintech, Wuhan, China) at 4°C overnight after rehydration. Subsequently, the sections were processed for detection with an anti-biotin system, followed by color development with 3,3′-diaminobenzidine (DAB) and counterstaining with hematoxylin. Ultimately, the sections were dehydrated, cleared, and coverslipped before being imaged using a scanner (SQS-12P, Shenzhen, China) at ×200 magnification. The images were then analyzed using ImageJ software.

### 2.10 Impact of TGF-β receptor 2 on phenotypic change in A549 cells

#### 2.10.1 Cell culture

Human lung cancer cells A549 were acquired from Procell (CL-0016). These cells were cultured in Ham’s F-12K medium (21127-022, Gibco, USA) supplemented with 10% FBS (10099-141C, Gibco, USA) and 1% antibody for both penicillin and streptomycin in a cell incubator (HF90, Heal Force, China) maintained at a temperature of 37°C and 5% CO_2_ atmosphere. Subsequently, the cells underwent trypsinization using 0.25% trypsin (25200072, Gibco, USA), and cells in the logarithmic growth phase were chosen for subsequent experiments.

#### 2.10.2 Cell transfection

Well-grown cells were seeded into six-well plates at a density of 70%–80% per well to ensure attachment to the bottom of the plates. After adhesion to the wells, the cells were randomly assigned to control group, si-NC group, and TGFBR2 siRNA (si-TGFBR2) group. Cells were transfected with jetPRIME transfection reagents (PT-114-75, Polyplus, France) as follows: 200 µL of jetPRIME buffer was added to an EP tube, to which siRNA at a final concentration of 15 nM was added. The contents were mixed by vortexing, and an additional 4 µL jetPRIME reagent was additional added. The resulting mixture was swirled and mixed thoroughly before being incubated at room temperature for 10 min. Finally, a 10-µL mixture was added to the culture fluid and were transferred into a fresh medium with 5% CO_2_ at 37°C, replacing the old media approximately 4–6 h later. Total RNA and total protein were extracted from LUAD cells 48 h after transfection, and transfection efficiency of cells was assessed using qPCR and Western blotting. To ensure efficient gene interference, two interference sequences targeting the TGFBR2 gene were designed, and the optimal sequence was selected for conducting subsequent assays. The sequences information of NC siRNA, TGFBR2 SIRNA-1, TGFBR2 SIRNA-2, TGFBR2, and GAPDH are shown in **Table 1**. Primers were synthesized by Beijing Tsingke Biotech Co., Ltd.

**Table 1:** Sequence information.

Name	Sequence(5′–3′)	Size
NC siRNA	UUCUCCGAACGUGUCACGUTT	
	TACGUGACACGUUCGGAGAATT	
TGFBR2 siRNA-1	GCAGAACACUUCAGAGCAGUUTT	
	AACUGCUCUGAAGUGUUCUGCTT	
TGFBR2 siRNA-2	CCUGUGUCGAAAGCAUGAAGGTT	
	CCUUCAUGCUUUCGACACAGGTT	
TGFBR2	AGCAGACCGATGTCTACTCCA	233 bp
	GCACTCAGTCAACGTCTCACA	
GAPDH	TCAAGAAGGTGGTGAAGCAGG	115 bp
	TCAAAGGTGGAGGAGTGGGT	

#### 2.10.3 Cell growth inhibition detected by cell counting Kit-8 assay

After 48 h of transfection, cells from each group were trypsinized to prepare a single-cell suspension, and the cell count was determined. Subsequently, the cells were seeded in a 5% CO_2_ atmosphere at 37°C for 0 h, 24 h, 48 h, and 72 h, respectively, with triplicate wells set up for each time point. A volume of 10 μL of CCK8 reagent (CK04, DOJINDO, Japan) was added to each well at each time point and incubated for another 3 h. Finally, the optical density (OD) at 450 nm was measured for each well using a microplate reader.

#### 2.10.4 Cell migration detected by Transwell assay

After transfection for 48 h, single-cell suspension was prepared through trypsinization, followed by cell counting to determine the cell number. The cell density of cells was adjusted to 1.5×10^5^/mL. A volume of 200 µL of the cell suspension was added to the Transwell chamber, while the lower chambers of the 24-well plates received 600 µL of cell medium supplemented with 10% FBS. Subsequently, the cells were incubated at 37°C for 24 h. Afterwards, the Transwell chamber was removed, and culture medium was discarded. The chamber was then washed twice with phosphate-buffered saline (PBS). Subsequently, the cells were fixed with 4% paraformaldehyde (PFA) for 10 min, stained with crystal violet, and washed twice in PBS after each step. A cotton swab was used to remove residual cells from the upper chambers. Cell migration was evaluated by counting the number of migrated tumor cells under a microscope in three random fields.

#### 2.10.5 Apoptosis detected by flow cytometry

At 48 h after transfection, the cells were digested with 0.25% trypsin, and all cells were collected for centrifugation at 200 g for 5 min at 4°C. The processed cells were collected for further use. These centrifuged cells were washed twice with pre-chilled PBS and centrifuged at 200 g each time for 5 min at 4°C. The obtained cells were resuspended in 100 μL 1× binding buffer after discarding the PBS, and 5 μL Annexin FITC and 2 μL PI (C035, GeneCodex, China) were added, and the mixture was gently mixed to ensure uniformity. Afterwards, the sample was incubated at room temperature, protected from light, for 15 min. Next, the sample was then placed on ice and analyzed by mixing the incubated solution with 400 μL 1× binding buffer and was placed on the ice for detection by a flow cytometer within 1 h.

### 2.11 Drug prediction and molecular docking

To identify potential small molecule drugs for treating LUAD, we predicted interactions between drugs and key genes associated with LUAD based on the Drug–Gene Interaction database (DGIDB). The predicted interactions were further analyzed using molecular docking techniques. Initially, we obtained the protein structure of the target genes from the Protein Data Bank (PDB) (27) and removed any small molecules and water molecules that were originally bound to the proteins. Protein hydrogenation and charge calculations were performed using the AutoDock tools. The drug structure was retrieved from PubChem database (28) and carried out charge-balanced, rotatable bond checks on small molecules using AutoDock Tools. Then, according to the receptor active center, the range of docking boxes was selected. AutoDock Vina was then used to calculate the receptor–ligand docking, and we selected the structure with the lowest binding free energy from the initial results for better sentence structure and clarity. Finally, PyMol software was employed for visualizing and enhancing the aesthetics of the docked structures.

## 3 Results

### 3.1 A total of 401 key module differential genes were associated with LUAD

**Figure 2:**
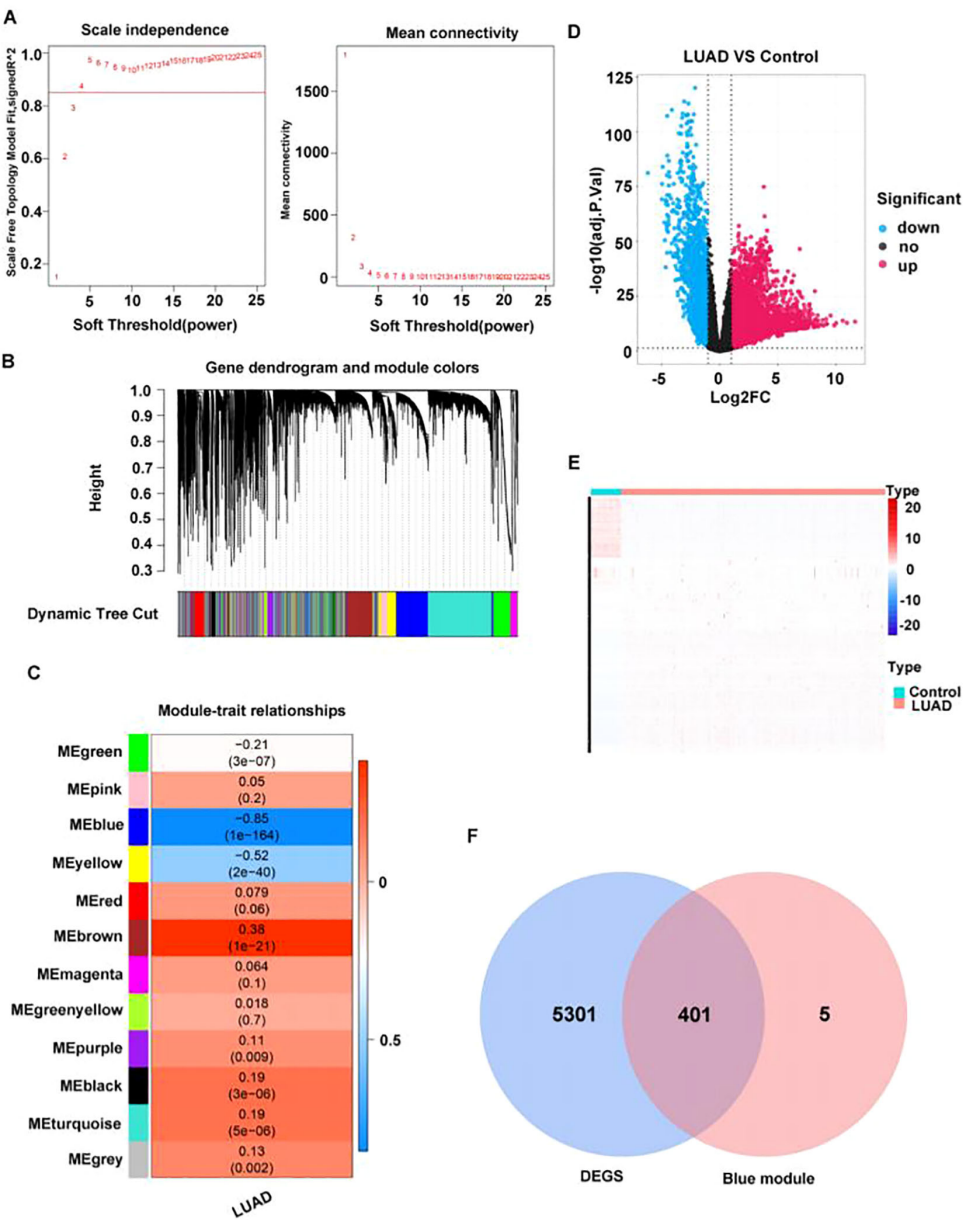
A total of 401 key module differential genes associated with LUAD were screened. **(A)** Selection of power values. **(B)** Module cluster tree diagram; the upper part is a hierarchical clustering tree diagram of genes, and the lower part is a gene module. **(C)** Heat map of the relationship between gene modules and traits based on LUAD. The darker the color, the higher the correlation, with red indicating a positive correlation and blue indicating a negative correlation. Numbers within cells indicate correlation and significance (correlations in the top row, p-values in the bottom row). **(D)** Volcano plot of differentially expressed genes between LUAD and normal groups. **(E)** Heat map of differentially expressed genes between LUAD and normal groups. Each row represents the relative expression level of a gene in all samples, each column represents the relative expression level of all genes in a sample, and the color of each square represents the relative expression level of the gene in the sample, with low expression in blue and high expression in red. **(F)** Venn diagram of differentially expressed genes in key modules.

In TCGA-LUAD, all samples were subjected to clustering analysis, which revealed no significant outliers (**Supplementary Figure S1**), R^2^ was set to 0.85, and the soft threshold β was determined to be 4 (**Figure 2A**) to build a scale-free network. Through WGCNA analysis, a total of 12 co-expression modules were identified (**Figure 2B**). Among these modules, the MEblue module exhibited the strongest negative correlation with LUAD (cor=−0.85, p=1×10^–164^) (**Figure 2C**).

Through analyzing the differential genes between LUAD and normal samples in TCGA dataset by the R package edgeR (version 3.36.0), 5,702 DEGs were identified, among which 3,785 were upregulated genes and 1,917 downregulated (**Figures 2D, E**). Then, the intersection between the 5702 DEGs and 406 key module genes was determined, resulting in 401 key module differential genes (**Figure 2F**).

### 3.2 Totally four candidate biomarkers were obtained through machine learning

**Figure 3:**
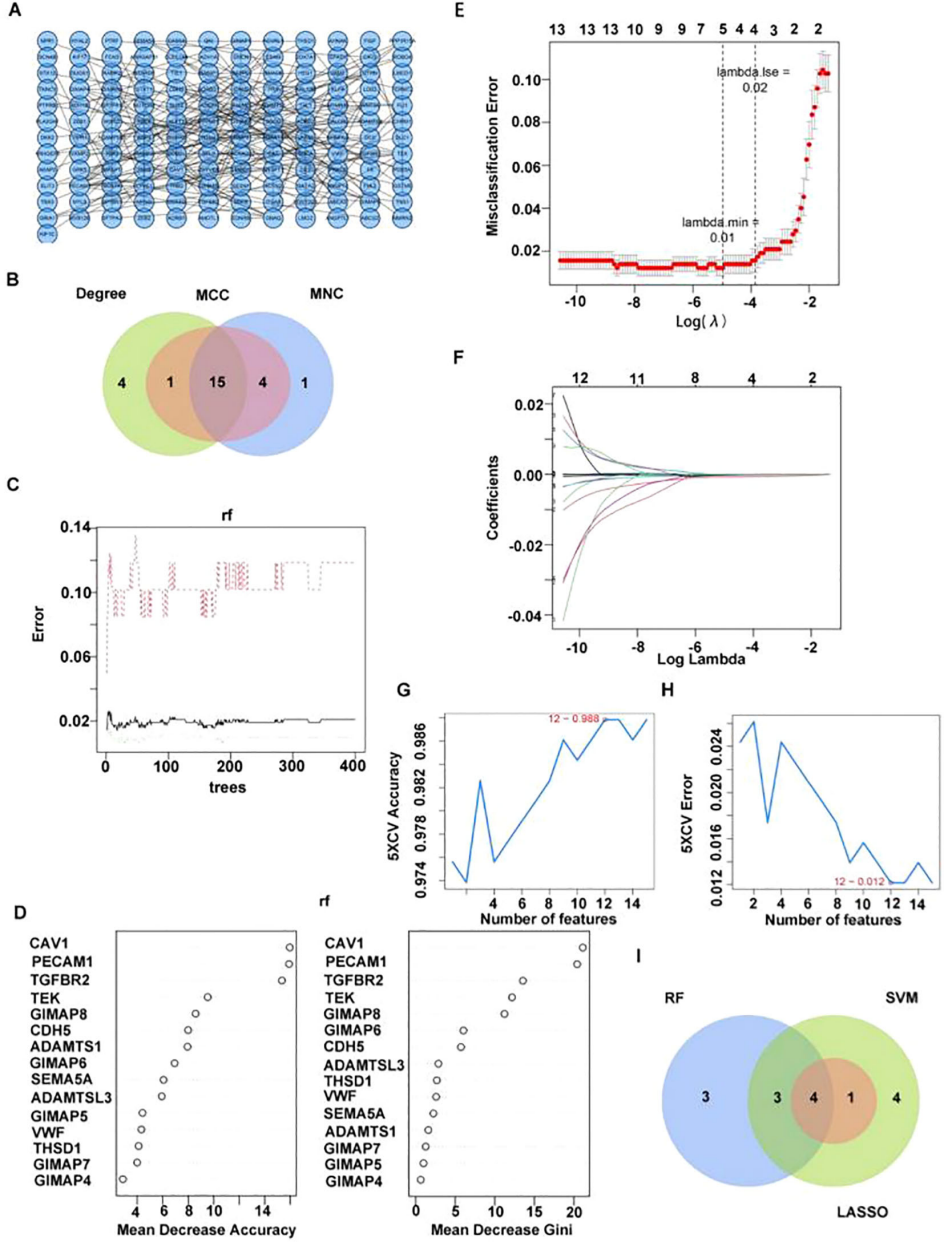
Four candidate biomarkers were obtained through machine learning. **(A)** PPI network diagram of key differentially expressed genes. **(B)** Venn diagram of candidate hub gene screened by three algorithms. **(C, D)** Identifying key candidate hub base maps through the random forest algorithm [**(C)** random forest error rate and number of classification trees; **D** ranking of gene importance]. **(E, F)** Screening key candidate hub genes through LASSO regression analysis. **(G, H)** Screening key genes through SVM-RFE [**(G)** Relationship diagram between prediction accuracy and feature number; **(H)** relationship diagram between generalization error and feature number]. **(I)** Venn diagram of candidate hub gene screened by three machine learning algorithms.

PPI network was constructed based on 401 key module differential genes (**Figure 3A**). Thereafter, an intersection analysis of the network’s topological parameters—MCC, MNC, and Degree—yielded 15 candidate genes (**Figure 3B**). The 15 candidate genes were then ranked, and the top 10 were selected as key candidate hub genes based on the Gini coefficient algorithm (mean decrease gini) (**Figures 3C, D**). The genes were analyzed through the application of the machine learning LASSO regression algorithm to screen five key candidate hub genes (**Figures 3E, F**). A total of 12 candidate hub genes were obtained using the SVM-RFE method (**Figures 3G, H**). Finally, the intersection of the genes obtained from the three machine learning algorithms was taken to obtain four hub genes (GIMAP6, CAV1, PECAM1, and TGFBR2) (**Figure 3I**).

### 3.3 All four candidate biomarkers had extremely strong predictive power

**Figure 4:**
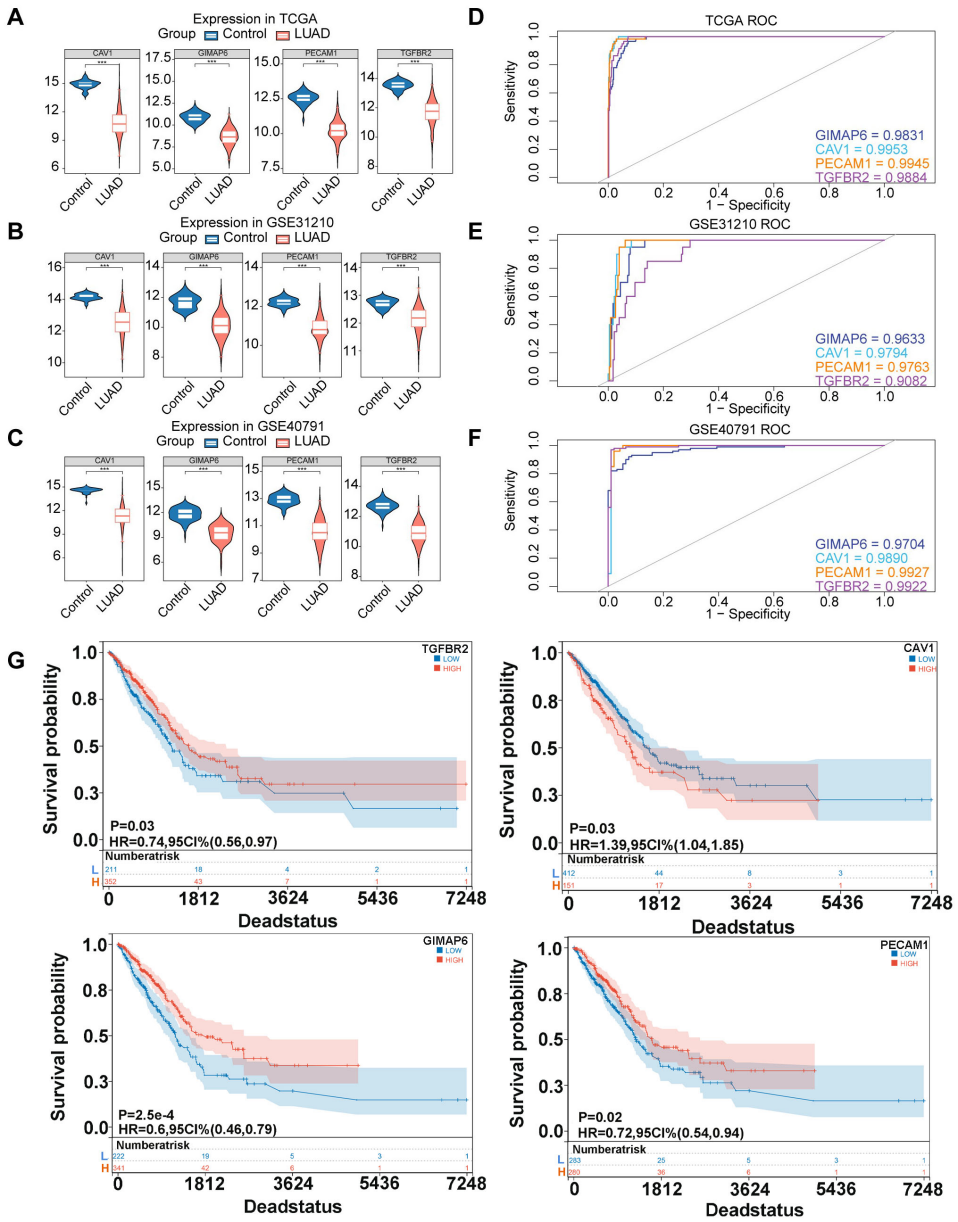
Expression, diagnosis, and survival analysis of four candidate biomarkers. **(A)** Expression of four hub genes in TCGA database. **(B)** Expression of four hub genes in GSE31210 database. **(C)** Expression of four hub genes in GSE40791 database. (Rank sum test was used for statistical analysis, ***p<0.001). **(D)** ROC curve chart of four hub genes in TGGA database. **(E)** ROC curve chart of four hub genes in GSE31210 database. **(F)** ROC curve chart of four hub genes in GSE40791 database. **(G)** K-M survival curves were plotted by log-rank test to investigate the effects of TGFBR2, CAV1, GIMAP6, and PECAM1 on the prognosis of LUAD patients.

In TCGA-LUAD, GSE40791, and GSE31210, it was observed that the expression levels of four candidate biomarkers were significantly lower in LUAD patients compared to controls (**Figures 4A–C**). Interestingly, ROC analysis revealed that these biomarkers had AUC values >0.9 across all datasets (TCGA-LUAD: GIMAP6, AUC=0.9831; CAV1, AUC=0.9953; PECAM1, AUC=0.9945; TGFBR2, AUC=0.9884; GSE31210: GIMAP6, AUC=0.9633; CAV1, AUC=0.9794; PECAM1, AUC=0.9763; TGFBR2, AUC=0.9082; GSE40791: GIMAP6, AUC= 0.9704;CAV1, AUC= 0.9890; PECAM1, AUC= 0.9927; TGFBR2, AUC= 0.9922), indicating their strong predictive power for LUAD (**Figures 4D–F**). Furthermore, K-M survival analysis demonstrated a significant association between the expression of these four biomarkers and patient prognosis (**Figure 4G**).

### 3.4 There was a causal relationship between TGFBR2 and LUAD in forward MR analysis

In assessing whether there is a causal relationship between gene expression and disease state, we used expression quantitative trait loci (eQTLs) as instrumental variables to infer causal associations between changes in gene expression and disease state. TGFBR2, GIMAP6, and CAV1 were considered as exposure factors, while LUAD was regarded as outcome. The TGFBR2-related dataset (eqtl-a-ENSG00000163513) included 31,470 samples and 19,824 SNPs. The GIMAP6-associated dataset (eqtl-a-ENSG00000133561) contained 31,684 samples and 20,006 SNPs, CAV1-related dataset (eqtl-a-ENSG00000105974) had 31,470 samples and 16,582 SNPs. Based on IVW results, only the relationship between eqtl-a-ENSG00000163513 (p=0.04, b=−0.06) and ieu-a-984 satisfied p < 0.05; however, the effect between eqtl-a-ENSG00000133561 (p=0.35) and ieu-a-984 satisfied p > 0.05, indicating that TGFBR2 has a significant causal relationship with LUAD, and it was a safe factor for LUAD.

**Figure 5:**
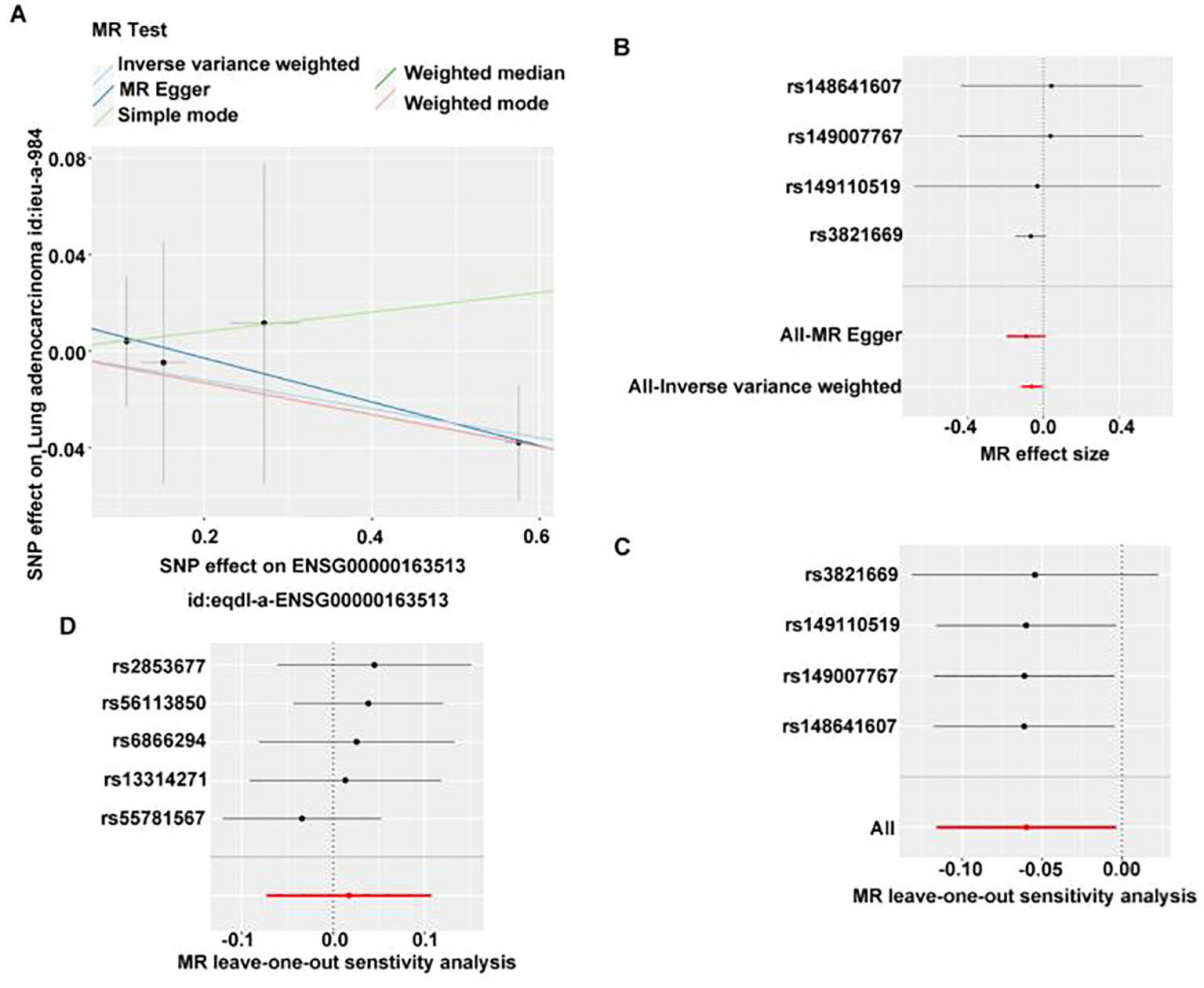
MR analysis of the causal relationship between TGFBR2 and LUAD. **(A)** Scatter plot of TGFBR2 SNP effect. **(B)** Forest map of TGFBR2 SNP. **(C)** Validation of TGFBR2 with leave-one-sensitivity analysis. **(D)** Reverse MR analysis of causal relationship between TGFBR2 and LUAD, validation of LUAD with leave-one-out sensitivity analysis.

In GWAS, we screened for SNPs significantly associated with LUAD (5×10^−8^, r^2^ = 0.001; kb=10,000) for IVW analysis. No causal effect was found for GIMAP6 and LUAD (**Supplementary Table S1**). Due to having fewer than three SNPs, the association between CAV1 and LUAD could not be evaluated using the IVW method. Subsequently, the scatter plot showed that the slope of IVW was negative and the intercept was negligible (**Figure 5A**). The further forest plot showed that the overall effect of IVW was <0 (**Figure 5B**). Therefore, TGFBR2 was the safety factor of LUAD. Afterwards, the reliability of MR analysis results was verified by sensitivity analysis. First, the Q_pval of the heterogeneity test was 0.949, indicating that there was no heterogeneity. Next, the p-value of the horizontal pleotropy test was 0.673, suggesting that no horizontal pleotropy (**Supplementary Table S2**). Finally, the residual SNPs had little effect on the whole after the single SNP was removed by the leave-one-out test, certificating that the MR results were reliable (**Figure 5C**).

Subsequently, we used lung adenocarcinoma as the exposure factor and TGFBR2 as the outcome. Screening of instrumental variables using 5×10^−8^, r^2^ = 0.001; kb=10,000 conditions yielded five instrumental variables associated with lung adenocarcinoma but not with TGFBR2 (**Supplementary Table S3**), IVW analysis revealed no significant causal effect of LUAD on TGFBR2 expression levels (p > 0.05) (**Supplementary Table S4**). Meanwhile, the p-value of horizontal pleiotropy was 0.494, indicating that there was no horizontal pleiotropy (**Supplementary Table S5**), and the leave-one-out test also indicated that the residual SNPs after removing a SNP had little effect on the overall effect (**Figure 5D**), which comprehensively indicated that the reverse MR analysis results were reliable. Finally, reverse MR analysis showed that LUAD did not lead to changes in TGFBR2 expression.

### 3.5 Relationship between TGFBR2 and clinical features of LUAD

**Figure 6:**
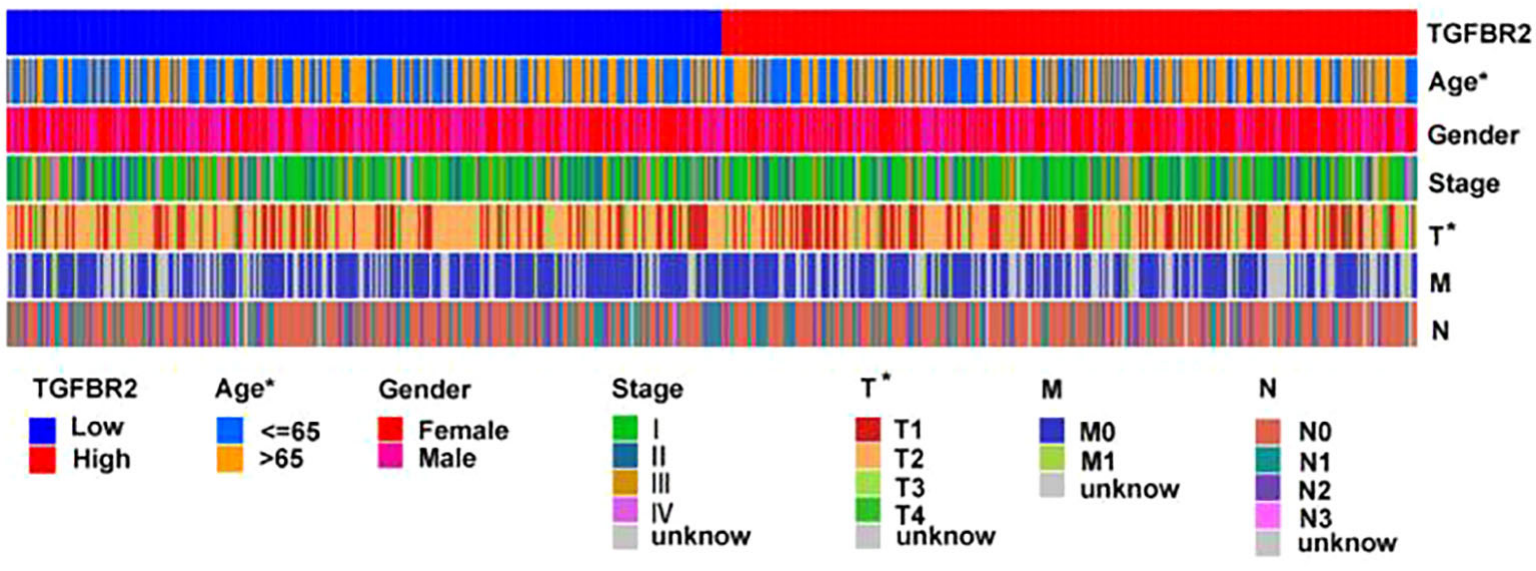
Relationship between TGFBR2 and clinical features of LUAD. (Rank-sum test was used for statistical analysis, *p<0.05).

To further explore the relationship between clinical features and TGFBR2 expression, we used data from LUAD samples from the TCGA-LUAD database to validate our findings. The results showed the TGFBR2 expression in relation to t-phase (T1, T2) and age in LUAD patients (**Figure 6**). The specific data and p-values for TGFBR2 across various clinical characteristics are presented in **Supplementary Figure S2**.

### 3.6 TGFBR2 and immune cell infiltration in LUAD

**Figure 7:**
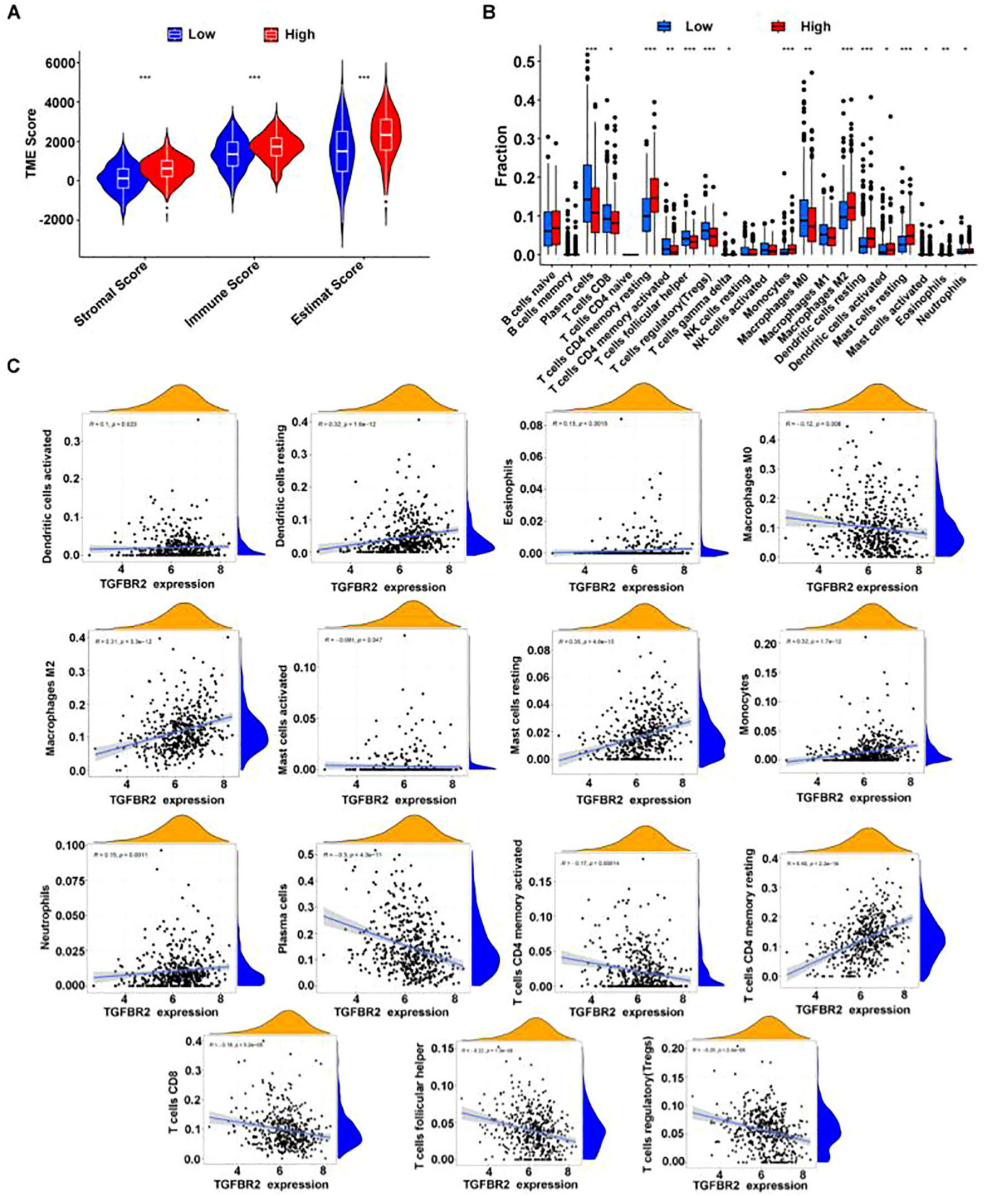
The correlation between TGFBR2 and immune infiltration. **(A)** Utilization estimates program assessment, immune infiltration parameters in groups with high and low TGFBR2 expression. **(B)** Immune cell infiltration analysis CIBERSORT deconvolution algorithm. (Rank-sum test was used for statistical analysis in (Panel **A**, **B**), *p<0.05, **p<0.01, ***p<0.001). **(C)** Scatter plot of the correlation between TGFBR2 expression and abundance of immune cell infiltration.

Utilizing the Estimate program to assess the immune microenvironment, the results indicate that the immune score is significantly higher in the high TGFBR2 expression group compared to the low expression group (**Figure 7A**). Analysis using the CIBERSORT algorithm reveals discrepancies in the proportion of 15 immune cell types, between high and low TGFBR2 expression groups (**Figure 7B**). Further correlation analysis shows that TGFBR2 expression is positively associated with the presence of dendritic cells, eosinophils, macrophages M2, mast cells, monocytes, neutrophils, and resting CD4 memory T cells, and negatively correlated with macrophages M0, plasma cells, T cells CD4 memory activated, T cells CD8, T cells follicular helper, and T cells regulatory (Tregs) (**Figure 7C**). The p-value = 0.047 between TGFBR2 and mast cells indicates that although this correlation is statistically significant, it is marginally close to the conventional threshold. These observations suggest a plausible connection link between TGFBR2 expression and immune infiltration in LUAD.

### 3.7 The expression of TGFBR2 was increased in LUAD tissue

**Figure 8:**
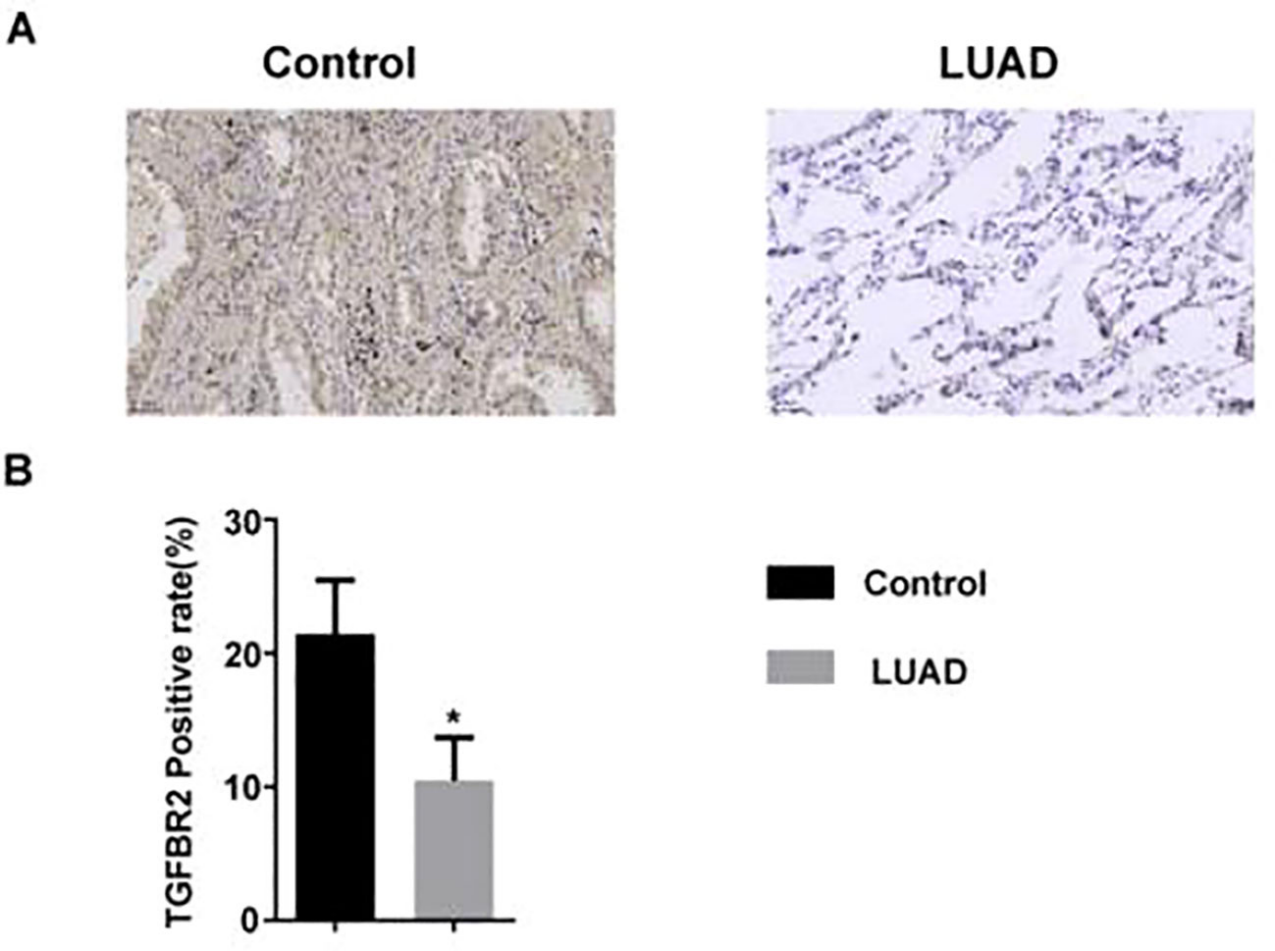
The expression of TGFBR2 in LUAD tissue. **(A)** Immunohistochemical staining of TGFBR2 in normal and LUAD tissues; scale bar=50 μm. **(B)** Analyzed in bar chart (mean ± SM, n=3 in control group, n=5 in LUAD group, t-test was used for statistical analysis, *p<0.05).

The expression level of the TGFBR2 in LUAD patients was measured by immunohistochemistry. The results showed that compared with normal lung tissues, TGFBR2 expression was decreased in LUAD tissues (**Figure 8**).

### 3.8 TGFBR2 siRNA promotes proliferation, migration, and invasion of lung cancer cell A549 and inhibits their apoptosis

#### 3.8.1 Expression of silenced TGFBR2

**Figure 9:**
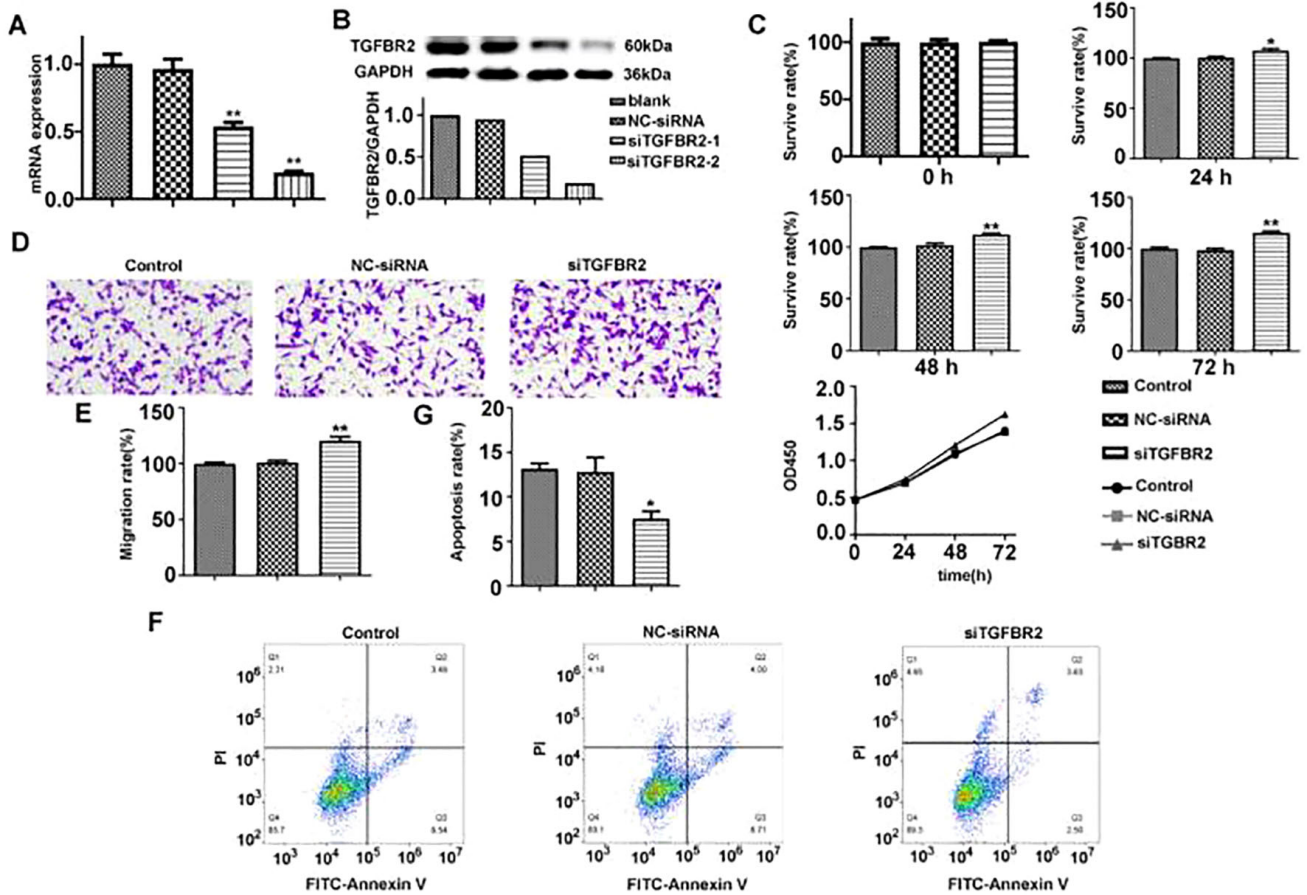
The effect of TGFBR2 on proliferation, migration, and apoptosis of A549 cells. **(A, B)** The transfection efficiency of siTGFBR2 was assessed by qRT-PCR and Western blotting. **(C)** The proliferation bar chart and proliferation curve of siTGFBR2 on A549 cells at three time points were detected by CCK8 assay. **(D)** Microscopic images of siTGFBR2 on A549 cells migration. **(E)** The bar chart of siTGFBR2 on A549 cells migration. **(F)** Flow cytometry analysis of siTGFBR2 on apoptosis of A549 cells. **(G)** The bar chart of siTGFBR2 on apoptosis of A549 cells. In bar charts, all values are represented by mean ± SM, and one-way ANOVA was used for statistical analysis, *p<0.05, **p<0.01.

To further verify the function of TGFBR2, we designed two siRNAs targeting TGFBR2 in this study and aimed to induce a quiescent or dormant state of the TGFBR2 gene in lung cancer cells. The transfection efficiency was assessed by qRT-PCR and Western blotting. The results showed that TGFBR2 expressions at both mRNA and protein levels in the siTGFBR2-1 and siTGFBR2-2 groups were decreased compared with the control group and the NC-siRNA group; this confirmed the success of the transfection assay, and the siTGFBR2-2 had better interference effect, making it suitable for use in subsequent experiments (**Figures 9A, B**).

#### 3.8.2 TGFBR2 siRNA promotes the proliferation of A549 cells

CCK8 assay was used to evaluate the effect of TGFBR2 siRNA on the proliferation of A549 cells. It revealed that A549 cells were increased in proliferation after siTGFBR2 transfection in a time-dependent manner. The siTGFBR2 group showed a significant increase in proliferation rate at the same time points when compared to the control group and the NC-siRNA group (p<0.01) (**Figure 9C**), suggesting that the TGFBR2 gene plays a role in inhibiting the proliferation of A549 cells.

#### 3.8.3 TGFBR2 siRNA promotes the migration of A549 cells

Transwell assay were performed to evaluate the effectiveness of TGFBR2 siRNA on the migration of A549 cells. The results showed that compared with the control group and the NC-siRNA group, the number of migrating cells in the siTGFBR2 group was notably increased (p<0.01) (**Figures 9D, E**). This finding suggests that the TGFBR2 gene plays a role in inhibiting the migration of A549 cells.

#### 3.8.4 TGFBR2 siRNA inhibits the apoptosis of A549 cells

Flow cytometry was used to evaluate the effect of TGFBR2 siRNA on the apoptosis of A549 cells. The results demonstrated that compared with the control group and the NC-siRNA group, the siTGFBR2 group showed a significantly decreased apoptosis rate (p<0.01) (**Figures 9F, G**), revealing that the TGFBR2 gene contributes to promoting apoptosis of A549 cells.

### 3.9 Molecular docking analyzes binding between drugs and protein

**Figure 10:**
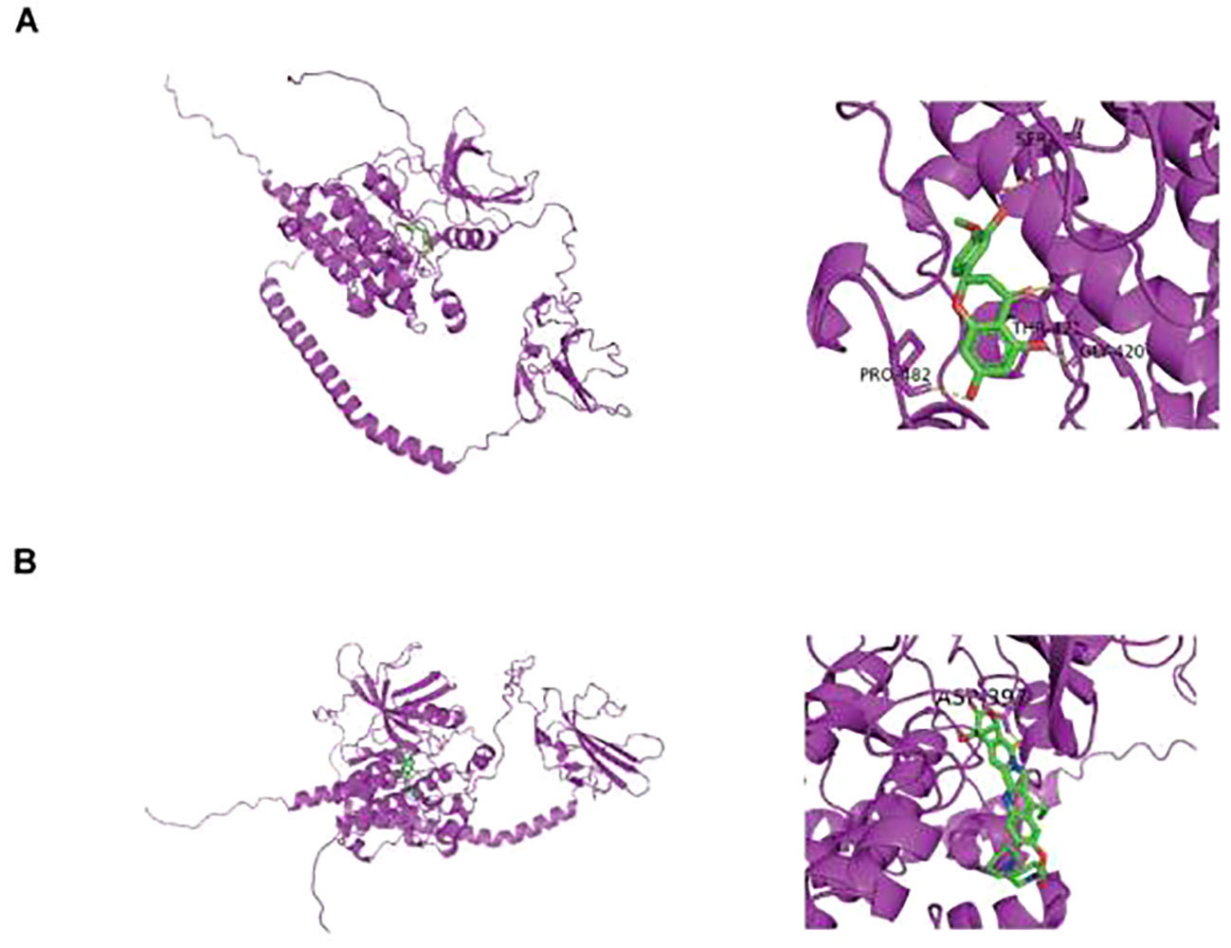
Prediction of small molecule drugs in TGFBR2. **(A)** Molecular docking of Irinotecan and TGFBR2. **(B)** Molecular docking of Hesperetin and TGFBR2.

In DGIDB database, two potential drugs targeting TGFBR2 were identified, Irinotecan and Hesperetin (**Table 2**). Then, the binding affinity between the drug and protein was analyzed by molecular docking. It was found that the binding affinity between Irinotecan and TGFBR2 was −8.7 kcal/mol, indicating that they had strong binding activity, while the binding affinity between Hesperetin and TGFBR2 was −6.2 kcal/mol, suggesting that they had graceful binding activity (**Figure 10**).

**Table 2:** Potential drug prediction targeting TGFBR2.

Gene	Drug	Sources	pmids
TGFBR2	IRINOTECAN	PharmGKB	27160286
TGFBR2	HESPERETIN	DTC	23153811

## 4 Discussion

LUAD is one of the most commonly occurring life-threatening malignancies, comprising roughly 40% of all lung cancer cases, and it has the highest incidence rate among all lung cancer subtypes (1). It has been elucidated that LUAD is involved in multi-gene process in its occurrence and development, and personalized or targeted therapy guided by these genetic testing results has emerged as primary treatment strategies for LUAD (29). Certain genes have been shown to exhibit strong associations with LUAD, and these discoveries have provided a basis for the development of chemotherapy and targeted drugs specific to LUAD, which have played an important role in delaying the progress of LUAD. In spite of these advances, there is still vast exploration for marker genes of the disease, and it is necessary to continue to further identify novel genes that affect the occurrence and development of LUAD, which is not only crucial for enhancing symptom management and extending patient survival but also of great significance for early screening, diagnosis, and treatment. As common approaches applied in bioinformatics, machine learning algorithms and Mendelian randomization technology provide convenience for the screening of disease markers at the technical level. In our study, we gathered data on LUAD patients from the TCGA and GEO databases, and employed the WGCNA algorithm to pinpoint 406 genes with significant associations with LUAD. We then utilized PPI networks and the algorithms MCC, MNC, and Degree, in conjunction with machine learning algorithms like random forest, LASSO regression, and SVM-RFE, to identify four hub genes: GIMAP6, CAV1, PECAM1, and TGFBR2. In the Kaplan–Meier analysis of four hub genes, CAV1 demonstrated a survival correlation that was opposite to that of GIMAP6, PECAM1, and TGFBR2. In LUAD, CAV1’s expression level and functional status are linked to various biological behaviors, such as the aggressiveness and metastatic capabilities of tumor cells. Higher expression levels of CAV1 may increase the invasiveness and migration of tumor cells, accelerating tumor progression and worsening patient outcomes (30). This observation aligns with previous studies, which have shown that high CAV1 expression correlates with a poorer prognosis in LUAD patients, as evidenced by significantly lower overall survival rates in the high-risk group compared to the low-risk group with low CAV1 expression. These findings collectively suggest an oncogenic role for CAV1 in LUAD progression.

Next, we conducted a single-variable MR analysis using these four genes as exposure variables and LUAD as an outcome. As a result, a causal relationship between TGFBR2 (cause) and lung adenocarcinoma (consequence) was found. Subsequently, immunohistochemical and cell experiments confirmed that TGFBR2 is a potential marker gene for LUAD. TGFBR2 serves as a pivotal component in the regulation of TGF-β pathway, and it encodes a transmembrane protein with a protein kinase domain that has the function of forming a heterodimeric complex with type 1 transforming growth factor β receptor and binds to TGF-β. The complex, upon receptor–ligand binding, phosphorylates proteins, which then enter the nucleus to regulate gene transcription associated with cell proliferation, cell cycle arrest, wound healing, immune suppression, and tumorigenesis (31). The previous studies revealed that TGF-β pathway has been shown to play a bidirectional adjusting effect in tumor progression: in the early stages of cancer, TGF-β exhibits tumor-suppressing effects by inhibiting cell cycle progression and promoting apoptosis; in advanced stage, TGF-β enables to promote tumor progression via accelerating tumor invasion and metastasis (32, 33). The role of TGFBR2 in tumors varies across various cancer types; for example, the expression of TGFBR2 increases in lung metastasis of osteosarcoma (34). Another study found that the expression levels of TGFBR2 gradually increased with the progression of hepatocarcinogenesis (35). It is believed that TGFBR2 plays a role involved in the occurrence and development of tumors, including NSCLC (36), pancreatic cancer (37), colorectal cancer (38), and liver cancer (39). At present, it is more widely recognized that TGFBR2 expression decreases in most NSCLC patients, and increasing TGFBR2 expression suppresses the proliferation and invasion of NSCLC (40, 41). Additionally, a study showed that the absence of TGFBR2 promotes the progression of lung squamous cell carcinoma (LUSC), which is also a common type of NSCLC (42). It is demonstrated that TGFBR2 is a key target for regulating NSCLC. However, some studies have proved that the mice with mutations in TGFBR2 have enhanced potential in tumor metastasis (43), and TGFBR2 receptor inhibitors enable reduced lung metastasis in squamous cell carcinoma (44). High expression of TGFBR2 in dominant NK cells of SCLC impairs their cytotoxic activity, leading to tumor growth and metastasis (45). In the study, we analyzed the expression of TGFBR2 in LUAD using the R graphics package, ggplot2, in the TCGA and GSE31210 datasets. The results showed that LUAD patients had decreased TGFBR2 expression, and MR analysis suggested that TGFBR2 is a protective factor for LUAD. The area under the ROC curve analysis for TGFBR2 in the TCGA and GSE31210 datasets was 0.9884 and 0.9084, respectively, suggesting that TGFBR2 possesses diagnostic value. Additionally, Kaplan–Meier analysis indicates that LUAD patients with high TGFBR2 expression experienced significantly prolonged overall survival. Immunohistochemical analysis of clinical tissue samples revealed that TGFBR2 expression was significantly lower in LUAD cancer tissues compared to controls. Functional validation of TGFBR2 demonstrated its capacity to inhibit proliferation and migration of A549 cells and to promote apoptosis. These findings align with both bioinformatics results and existing literature.

The tumor microenvironment (TME) is critical in the initiation and progression of cancer, with immune cells playing a vital role within this environment. In the TME, immune cell infiltration can act both as an immune surveillance mechanism to hinder tumor progression and as a promoter of tumor-favorable phenotypes, facilitating immune escape and further tumor development. Consequently, targeting immune cells has become a pivotal approach in tumor immunotherapy. In this study, analysis of immune cell infiltration algorithms revealed that levels of infiltration by dendritic cells, eosinophils, macrophages, neutrophils, CD4+ T cells, and Treg cells correlate with TGFBR2 expression. Notably, positive correlations were found with dendritic cells, CD4+ T cells, and neutrophils, suggesting that TGFBR2 may play a significant role in enhancing the immune regulatory network within the LUAD tumor microenvironment. Research suggested that as a molecule with immune-regulatory functions, high expression of TGFBR2 can trigger innate immune responses and mitigate immune suppression, facilitating T-cell activation and infiltration, and thereby curbing tumor growth (46). However, further studies are necessary to confirm the link between TGFBR2 expression and immune infiltration in LUAD. The outcomes of this study offer valuable insights into the potential role of TGFBR2 in immunotherapy for LUAD and may aid in the development of personalized immunotherapy strategies.

In our study, two drugs (Irinotecan and Hesperetin) targeting TGFBR2 through molecular docking were predicted after determining correlation between TGFBR2 and LUAD. Irinotecan, an anti-tumor enzyme inhibitor, is widely used in treating colorectal cancer and is also approved for application in treatment of pancreatic cancer, lung cancer, and other cancers (47). A series of studies have shown that SCLC patients receiving Irinotecan for maintenance chemotherapy have a longer survival (48). Additionally, it is also used in chemotherapy regimens for advanced non-small-cell lung cancer and recurrent NSCLC (49). The results of this study suggested that Irinotecan has potential advantages in treating LUAD patients with abnormal TGFBR2 expression. Hesperetin, a naturally active bio-compound, belongs to one of flavanones. A series of studies have shown that Hesperetin is functioning in inhibiting the proliferation and migration of LUAD cells and is capable of restraining tumor growth in LUAD mouse models combining with use of carboplatin (50). Therefore, Hesperetin is of important value in research and application for treating lung cancer, especially for LUAD patients with abnormal TGFBR2 expressions.

Although this study highlights the potential of TGFBR2 as a protective biomarker in lung adenocarcinoma (LUAD), it does have certain limitations. First, the reliance primarily on the TCGA-LUAD, GSE31210, and GSE40791 datasets may compromise the universality and representativeness of the results. Future research should utilize larger-scale, multi-center data to increase the reliability of these findings. Second, the research validated the function of TGFBR2 using the A549 cell line, but it is important to note that cell characteristics and experimental conditions *in vitro* can differ from those *in vivo*. Additionally, the drugs Irinotecan and Hesperetin, which were identified as potentially related to TGFBR2 through the DGIDB, require validation for safety and efficacy in preclinical and clinical trials. In conclusion, while this study provides initial evidence supporting TGFBR2’s role in LUAD, addressing these limitations is essential for its effective application as a biomarker and therapeutic target. Future research should concentrate on expanding sample sizes, exploring mechanisms in greater depth, and validating drug predictions to advance the use of TGFBR2 in treating LUAD.

## 5 Conclusions

In this study, we tried to accurately screen genes closely relating to diagnosis and prognosis of LUAD based on the WGCNA and bioinformatic methods including machine learning algorithms and MR. In addition, TGFBR2 is verified as a potential diagnostic and prognostic marker for LUAD via immunohistochemistry and cell experiments. It is related to LUAD immune cell infiltration, which can be a potential target for LUAD immunotherapy. Furthermore, Irinotecan and Hesperetin are predicted as potential drugs targeting LUAD, but further experiments and clinical trials are needed for verification. The study provides some insights for the diagnosis and treatment of LUAD. In the future study, we will continue to focus on the role of this gene in LUAD and conduct more studies on LUAD-related markers.

## Data Availability

The original contributions presented in the study are included in the article/**Supplementary Material**. Further inquiries can be directed to the corresponding authors.
