# Supplementary material for: Identification of potential biomarkers for lung adenocarcinoma: a study based on bioinformatics analysis combined with validation experiments
Source: Front Oncol. 2024 Sep 19;14:1425895. doi: 10.3389/fonc.2024.1425895 (PMC11446723; doi:10.3389/fonc.2024.1425895)
Supplement: Supplementary file 1 [file DataSheet1.zip › Data Sheet 2/Supplementary Fig.2.pdf]

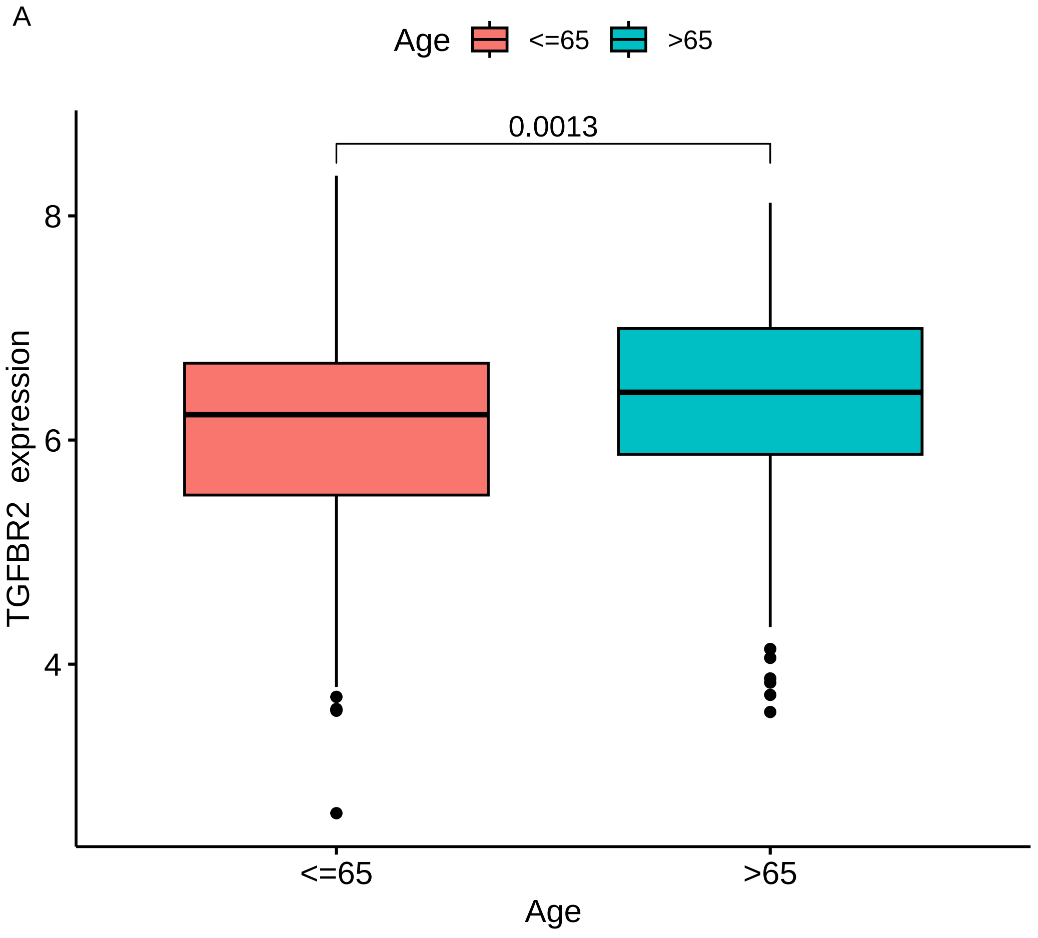
A
Age
<=65
>65
0.0013
8
6
4
TGFBR2 expression
<=65
>65
Age

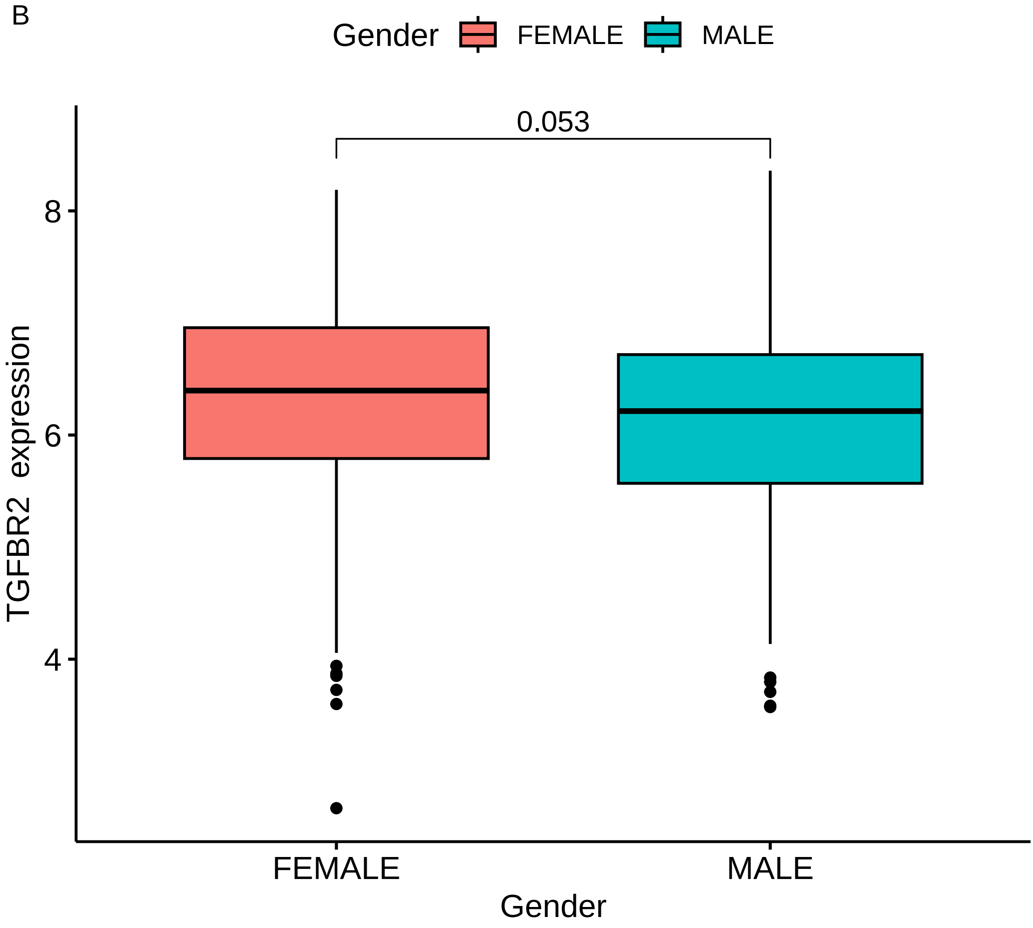
B
Gender
FEMALE
MALE
0.053
8
6
4
TGFBR2 expression
FEMALE
MALE
Gender

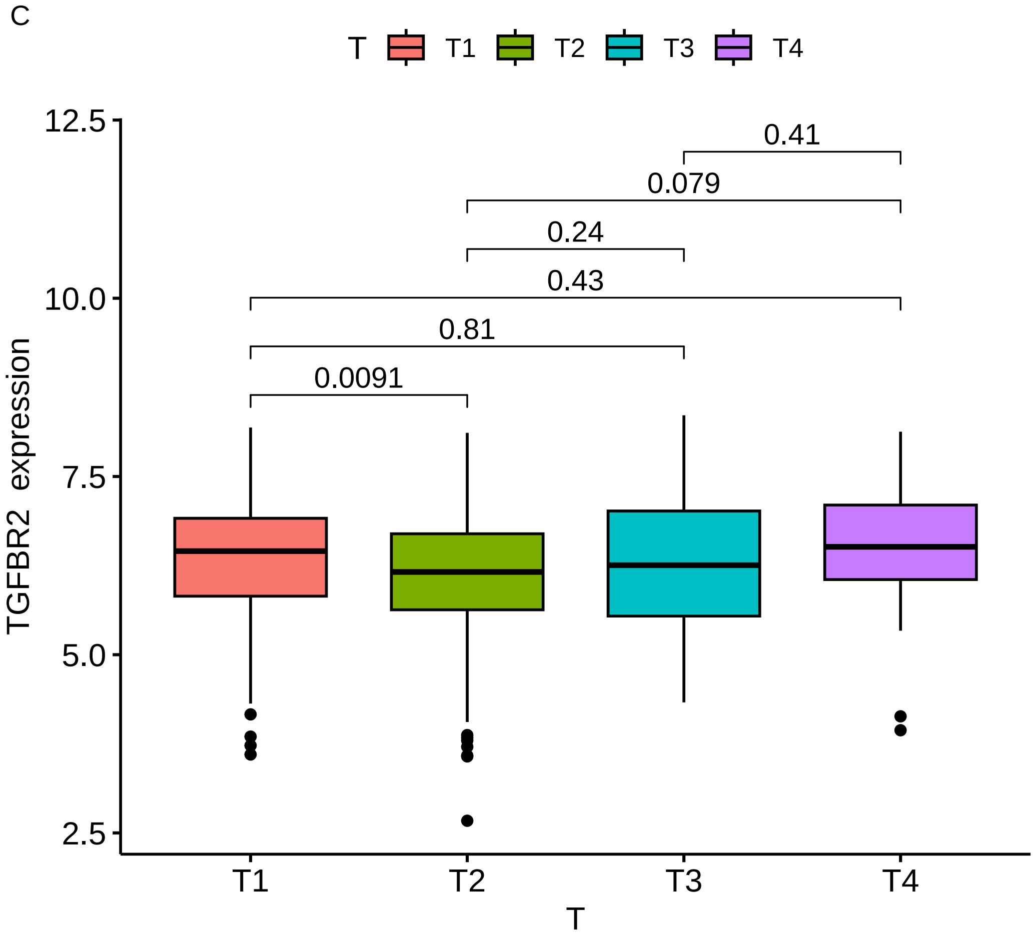
C
T
T1
T2
T3
T4
0.41
0.079
0.24
0.43
0.81
0.0091
12.5
10.0
7.5
5.0
2.5
TGFBR2 expression
T1
T2
T3
T4
T

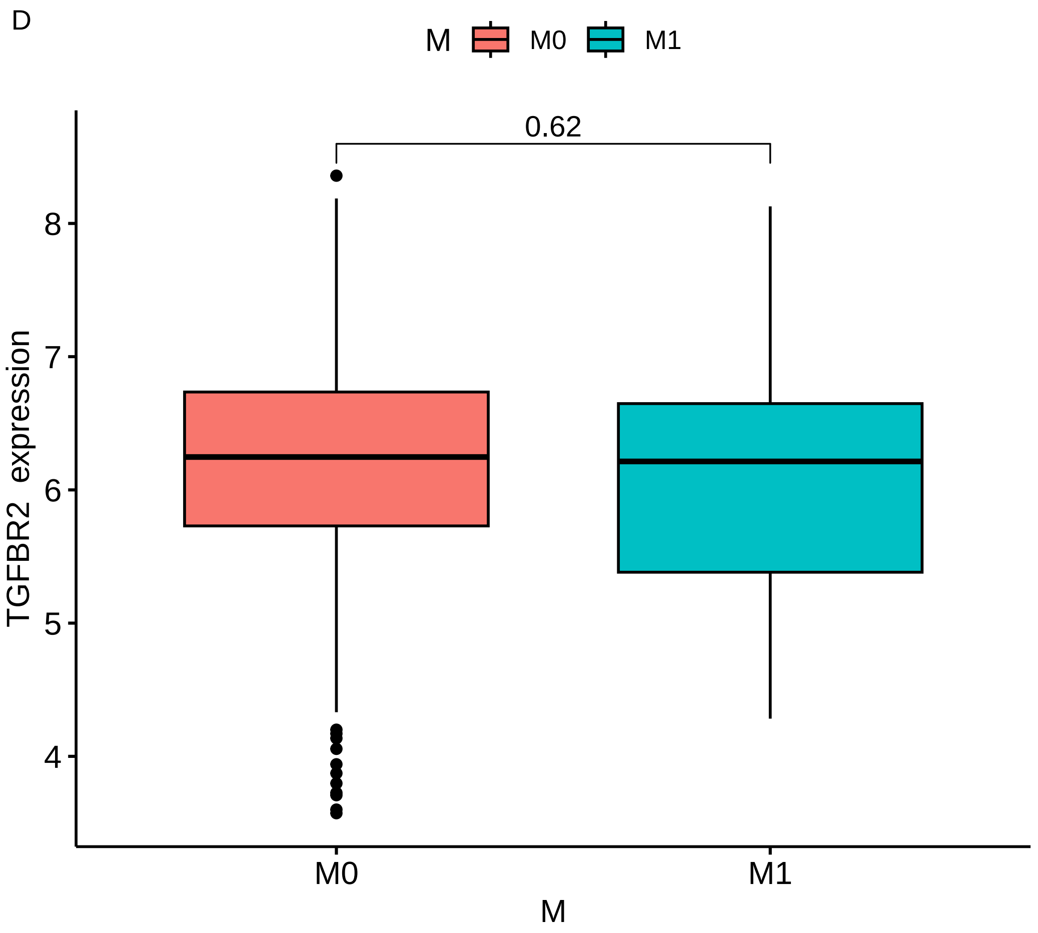
D
M
M0
M1
0.62
TGFBR2_expression
8
7
6
5
4
M0
M1
M

E

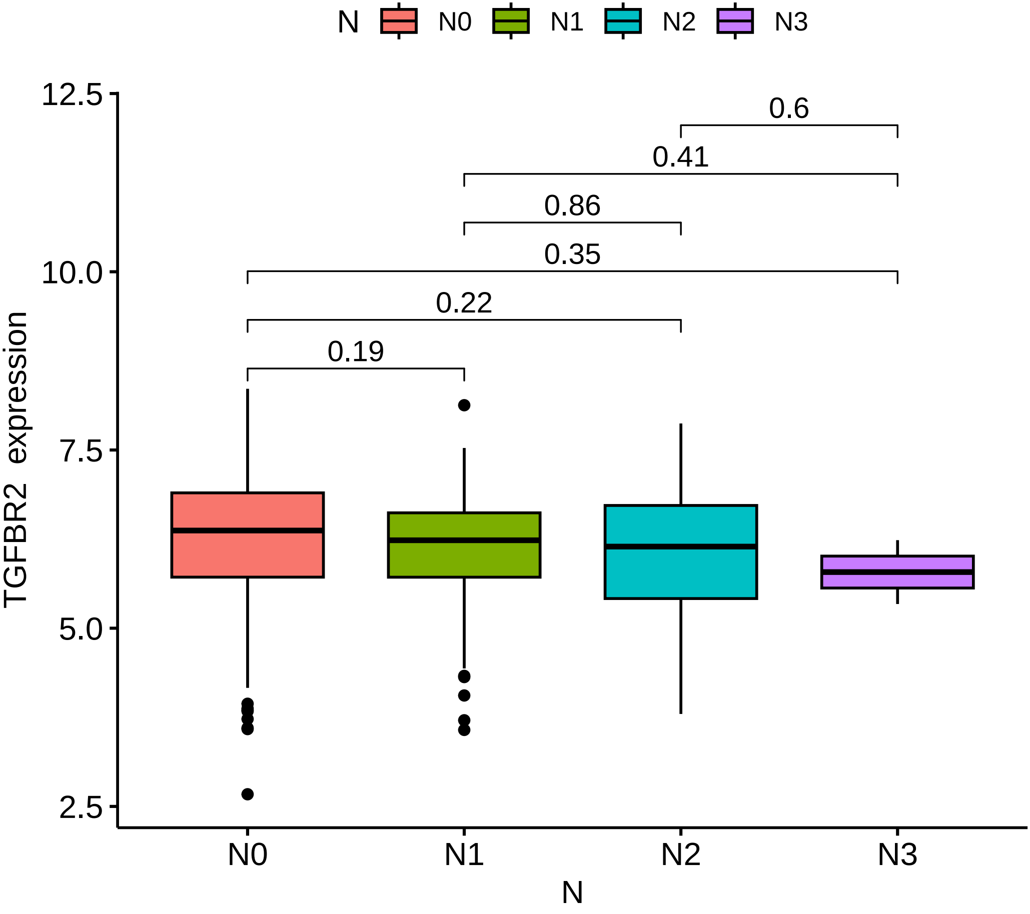

N
N0
N1
N2
N3
0.6
0.41
0.86
0.35
0.22
0.19
12.5
10.0
7.5
5.0
2.5
TGFBR2 expression
N0
N1
N2
N3
N

F

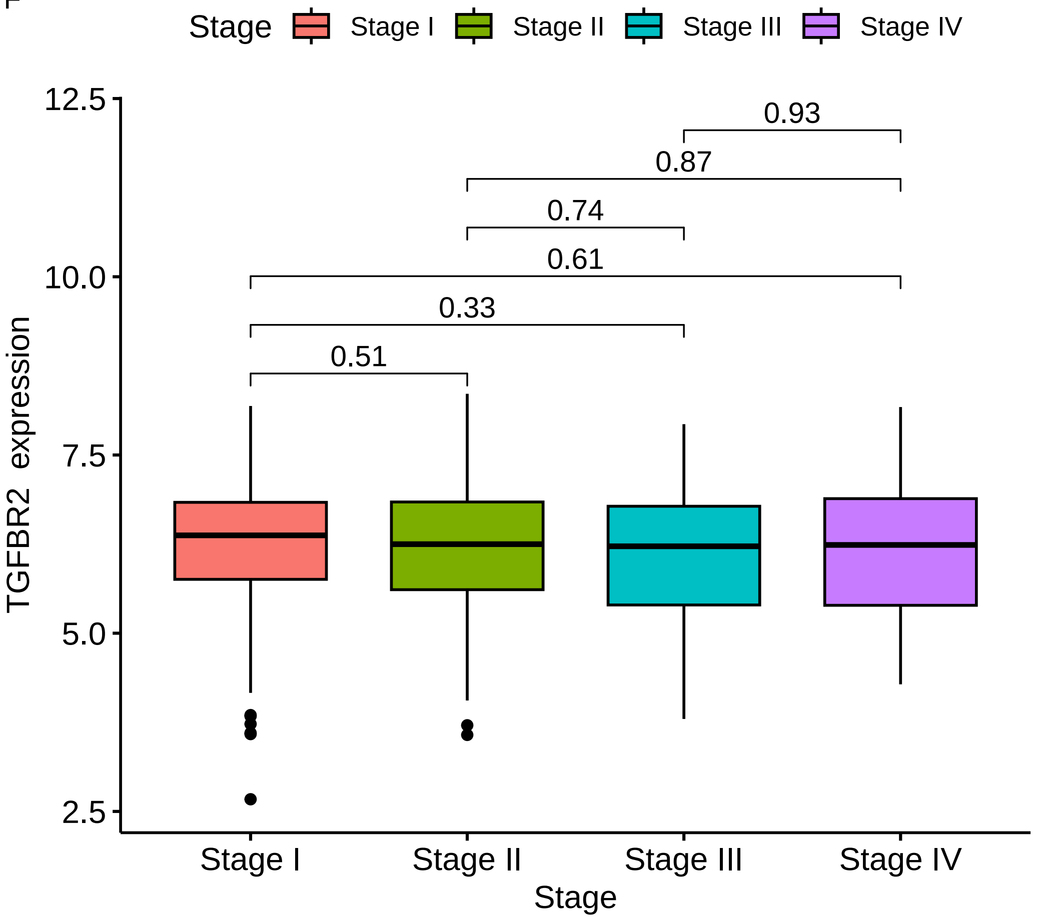
Stage
Stage I
Stage II
Stage III
Stage IV
0.93
0.87
0.74
0.61
0.33
0.51
12.5
10.0
7.5
5.0
2.5
TGFBR2 expression
Stage I
Stage II
Stage III
Stage IV
Stage

Supplementary Fig.2 Relationship Between TGFBR2 and Clinical Features of LUAD.

A Relationship between TGFBR2 and age of LUAD patients.

B Relationship between TGFBR2 and gender of LUAD patients.

C-E Relationship between TGFBR2 and TNM classification of LUAD.

F Relationship between TGFBR2 and stage of LUAD.
