# Supplementary material for: Identification of potential biomarkers for lung adenocarcinoma: a study based on bioinformatics analysis combined with validation experiments
Source: Front Oncol. 2024 Sep 19;14:1425895. doi: 10.3389/fonc.2024.1425895 (PMC11446723; doi:10.3389/fonc.2024.1425895)
Supplement: Supplementary file 1 [file DataSheet1.zip › Data Sheet 2/supplementary table/Supplementary Table1.docx]

Supplementary Table1 Mendelian analysis of exposure factors and outcomes

| id.exposure | id.outcome | outcome | exposure | method | nsnp | b | se | pval | b_direction | p_no |
| --- | --- | --- | --- | --- | --- | --- | --- | --- | --- | --- |
| eqtl-a-ENSG00000133561 | ieu-a-984 | Lung adenocarcinoma \|\| id:ieu-a-984 | ENSG00000133561 \|\| id:eqtl-a-ENSG00000133561 | MR Egger | 3 | 0.0100205 | 0.0894585 | 0.929 |  | MR Egger Weighted median IVW |
| eqtl-a-ENSG00000133561 | ieu-a-984 | Lung adenocarcinoma \|\| id:ieu-a-984 | ENSG00000133561 \|\| id:eqtl-a-ENSG00000133561 | Weighted median | 3 | 0.0122778 | 0.0395581 | 0.756 |  | MR Egger Weighted median IVW |
| eqtl-a-ENSG00000133561 | ieu-a-984 | Lung adenocarcinoma \|\| id:ieu-a-984 | ENSG00000133561 \|\| id:eqtl-a-ENSG00000133561 | Inverse variance weighted (multiplicative random effects) | 3 | 0.0440068 | 0.0466482 | 0.345 |  | MR Egger Weighted median IVW |
| eqtl-a-ENSG00000163513 | ieu-a-984 | Lung adenocarcinoma \|\| id:ieu-a-984 | ENSG00000163513 \|\| id:eqtl-a-ENSG00000163513 | MR Egger | 4 | -0.090791 | 0.0528764 | 0.1466198 |  | MR Egger Weighted median |
| eqtl-a-ENSG00000163513 | ieu-a-984 | Lung adenocarcinoma \|\| id:ieu-a-984 | ENSG00000163513 \|\| id:eqtl-a-ENSG00000163513 | Weighted median | 4 | -0.065793 | 0.0317663 | 0.038345 |  | MR Egger Weighted median |
| eqtl-a-ENSG00000163513 | ieu-a-984 | Lung adenocarcinoma \|\| id:ieu-a-984 | ENSG00000163513 \|\| id:eqtl-a-ENSG00000163513 | Inverse variance weighted (multiplicative random effects) | 4 | -0.059814 | 0.02863 | 0.0366879 |  | MR Egger Weighted median |
| eqtl-a-ENSG00000105974 | ieu-a-984 | Lung adenocarcinoma \|\| id:ieu-a-984 | ENSG00000105974 \|\| id:eqtl-a-ENSG00000105974 | Inverse variance weighted (multiplicative random effects) | 1 | 0.0209509 | NaN | NaN |  | MR Egger Weighted median IVW |
