# Supplementary material for: Identification of potential biomarkers for lung adenocarcinoma: a study based on bioinformatics analysis combined with validation experiments
Source: Front Oncol. 2024 Sep 19;14:1425895. doi: 10.3389/fonc.2024.1425895 (PMC11446723; doi:10.3389/fonc.2024.1425895)
Supplement: Supplementary file 1 [file DataSheet1.zip › Data Sheet 2/supplementary table/Supplementary Table2.docx]

Supplementary Table2-1 Heterogeneity test of Mendelian analysis between TGFBR2 and lung adenocarcinoma

| id.exposure | id.outcome | outcome | exposure | method | Q | Q_df | Q_pval |
| --- | --- | --- | --- | --- | --- | --- | --- |
| ieu-a-984 | eqtl-a-ENSG00000163513 | ENSG00000163513 \|\| id:eqtl-a-ENSG00000163513 | Lung adenocarcinoma \|\| id:ieu-a-984 | MR Egger | 7.0827436 | 2 | 0.0289736 |
| ieu-a-984 | eqtl-a-ENSG00000163513 | ENSG00000163513 \|\| id:eqtl-a-ENSG00000163513 | Lung adenocarcinoma \|\| id:ieu-a-984 | Inverse variance weighted | 9.5156461 | 3 | 0.0231655 |

Supplementary Table2-2 Horizontal pleiotropy test of Mendelian analysis between TGFBR2 and lung adenocarcinoma

| id.exposure | id.outcome | outcome | exposure | egger_intercept | se | pval |
| --- | --- | --- | --- | --- | --- | --- |
| ieu-a-984 | eqtl-a-ENSG00000163513 | ENSG00000163513 \|\| id:eqtl-a-ENSG00000163513 | Lung adenocarcinoma \|\| id:ieu-a-984 | -0.031679319 | 0.038220755 | 0.494357924 |
