# Supplementary material for: Identification of potential biomarkers for lung adenocarcinoma: a study based on bioinformatics analysis combined with validation experiments
Source: Front Oncol. 2024 Sep 19;14:1425895. doi: 10.3389/fonc.2024.1425895 (PMC11446723; doi:10.3389/fonc.2024.1425895)
Supplement: Supplementary file 1 [file DataSheet1.zip › Data Sheet 2/supplementary table/Supplementary Table3.docx]

Supplementary Table3 Five SNPs were associated with LUAD

| SNP | effect_allele.  exposure | other_allele.  exposure | effect_allele.  outcome | other_allele.  outcome | beta.exposure | beta.outcome | eaf.exposure | eaf.outcome |
| --- | --- | --- | --- | --- | --- | --- | --- | --- |
| rs13314271 | C | T | C | T | -0.114672 | -0.006541 | 0.49468 | 0.490772 |
| rs2853677 | A | G | A | G | -0.205652 | 0.0116785 | 0.575827 | 0.588324 |
| rs55781567 | G | C | G | C | 0.236491 | 0.0285734 | 0.357607 | 0.329524 |
| rs56113850 | C | T | C | T | 0.120742 | -0.022058 | 0.554742 | 0.565098 |
| rs6866294 | A | T | C | T | -0.150881 | -0.004801 | 0.408136 | 0.59428 |

| remove | palindromic | ambiguous | id.outcome | chr | pos | se.outcome | samplesize.outcome | pval.outcome |
| --- | --- | --- | --- | --- | --- | --- | --- | --- |
| FALSE | FALSE | FALSE | eqtl-a-ENSG00000163513 | 3 | 189357602 | 0.0119054 | 26280 | 0.582731 |
| FALSE | FALSE | FALSE | eqtl-a-ENSG00000163513 | 5 | 1287194 | 0.0120933 | 18405 | 0.334144 |
| FALSE | TRUE | FALSE | eqtl-a-ENSG00000163513 | 15 | 78857986 | 0.0126599 | 31385 | 0.0240082 |
| FALSE | FALSE | FALSE | eqtl-a-ENSG00000163513 | 19 | 41353107 | 0.0120042 | 29493 | 0.0661515 |
| TRUE | TRUE | FALSE | eqtl-a-ENSG00000163513 | 5 | 1315660 | 0.0121209 | 26801 | 0.692105 |

| remove | palindromic | ambiguous | id.outcome | chr | pos | se.outcome | samplesize.outcome | pval.outcome |
| --- | --- | --- | --- | --- | --- | --- | --- | --- |
| FALSE | FALSE | FALSE | eqtl-a-ENSG00000163513 | 3 | 189357602 | 0.0119054 | 26280 | 0.582731 |
| FALSE | FALSE | FALSE | eqtl-a-ENSG00000163513 | 5 | 1287194 | 0.0120933 | 18405 | 0.334144 |
| FALSE | TRUE | FALSE | eqtl-a-ENSG00000163513 | 15 | 78857986 | 0.0126599 | 31385 | 0.0240082 |
| FALSE | FALSE | FALSE | eqtl-a-ENSG00000163513 | 19 | 41353107 | 0.0120042 | 29493 | 0.0661515 |
| TRUE | TRUE | FALSE | eqtl-a-ENSG00000163513 | 5 | 1315660 | 0.0121209 | 26801 | 0.692105 |

| outcome | originalname.  outcome | outcome.  deprecated | mr_keep.  outcome | | data_source.  outcome | proxy.  outcome | target_  snp.outcome | proxy_  snp.outcome |
| --- | --- | --- | --- | --- | --- | --- | --- | --- |
| ENSG00000163513 \|\| id:eqtl-a-ENSG00000163513 | ENSG00000163513 | ENSG00000163513 \|\| \|\| | TRUE | igd | | NA | NA | NA |
| ENSG00000163513 \|\| id:eqtl-a-ENSG00000163513 | ENSG00000163513 | ENSG00000163513 \|\| \|\| | TRUE | igd | | NA | NA | NA |
| ENSG00000163513 \|\| id:eqtl-a-ENSG00000163513 | ENSG00000163513 | ENSG00000163513 \|\| \|\| | TRUE | igd | | NA | NA | NA |
| ENSG00000163513 \|\| id:eqtl-a-ENSG00000163513 | ENSG00000163513 | ENSG00000163513 \|\| \|\| | TRUE | igd | | NA | NA | NA |
| ENSG00000163513 \|\| id:eqtl-a-ENSG00000163513 | ENSG00000163513 | ENSG00000163513 \|\| \|\| | TRUE | igd | | TRUE | rs6866294 | rs4975616 |

| target_  a1.outcome | target_  a2.outcome | proxy_  a1.outcome | proxy_  a2.outcome | se.exposure | chr.exposure | | pos.exposure | | pval.exposure | samplesize.exposure |
| --- | --- | --- | --- | --- | --- | --- | --- | --- | --- | --- |
| NA | NA | NA | NA | 0.016237 | 3 | 189357602 | | 1.64E-12 | | 65864 |
| NA | NA | NA | NA | 0.017069 | 5 | 1287194 | | 1.98E-33 | | 65864 |
| NA | NA | NA | NA | 0.016749 | 15 | 78857986 | | 2.89E-45 | | 65864 |
| NA | NA | NA | NA | 0.01871 | 19 | 41353107 | | 1.09E-10 | | 60770 |
| T | C | G | A | 0.016577 | 5 | 1311693 | | 8.89E-20 | | 65864 |

| id.exposure | exposure | mr_keep.  exposure | pval_origin.  exposure | data_source.  exposure | action | mr_keep | R2 | F |
| --- | --- | --- | --- | --- | --- | --- | --- | --- |
| ieu-a-984 | Lung adenocarcinoma \|\| id:ieu-a-984 | TRUE | reported | igd | 2 | TRUE | 0.0065741 | 435.84798 |
| ieu-a-984 | Lung adenocarcinoma \|\| id:ieu-a-984 | TRUE | reported | igd | 2 | TRUE | 0.02066 | 1389.4162 |
| ieu-a-984 | Lung adenocarcinoma \|\| id:ieu-a-984 | TRUE | reported | igd | 2 | TRUE | 0.025696 | 1737.0267 |
| ieu-a-984 | Lung adenocarcinoma \|\| id:ieu-a-984 | TRUE | reported | igd | 2 | TRUE | 0.0072019 | 477.77509 |
| ieu-a-984 | Lung adenocarcinoma \|\| id:ieu-a-984 | TRUE | reported | igd | 2 | FALSE | 0.0109983 | 732.4261 |
