# Supplementary material for: Identification of potential biomarkers for lung adenocarcinoma: a study based on bioinformatics analysis combined with validation experiments
Source: Front Oncol. 2024 Sep 19;14:1425895. doi: 10.3389/fonc.2024.1425895 (PMC11446723; doi:10.3389/fonc.2024.1425895)
Supplement: Supplementary file 1 [file DataSheet1.zip › Data Sheet 2/supplementary table/supplementary Table4.docx]

Supplementary Table4 Reverse Mendelian randomization analysis

| id.exposure | id.outcome | outcome | exposure | method | nsnp | b | se | pval |
| --- | --- | --- | --- | --- | --- | --- | --- | --- |
| ieu-a-984 | eqtl-a-ENSG00000163513 | ENSG00000163513 \|\| id:eqtl-a-ENSG00000163513 | Lung adenocarcinoma \|\| id:ieu-a-984 | MR Egger | 5 | 0.1874984 | 0.2180093 | 0.4803961 |
| ieu-a-984 | eqtl-a-ENSG00000163513 | ENSG00000163513 \|\| id:eqtl-a-ENSG00000163513 | Lung adenocarcinoma \|\| id:ieu-a-984 | Weighted median | 5 | 0.0448453 | 0.0489519 | 0.35961 |
| ieu-a-984 | eqtl-a-ENSG00000163513 | ENSG00000163513 \|\| id:eqtl-a-ENSG00000163513 | Lung adenocarcinoma \|\| id:ieu-a-984 | Inverse variance weighted | 5 | 0.0150813 | 0.0617412 | 0.8070246 |
| ieu-a-984 | eqtl-a-ENSG00000163513 | ENSG00000163513 \|\| id:eqtl-a-ENSG00000163513 | Lung adenocarcinoma \|\| id:ieu-a-984 | Simple mode | 5 | 0.047367 | 0.1020798 | 0.674231 |
| ieu-a-984 | eqtl-a-ENSG00000163513 | ENSG00000163513 \|\| id:eqtl-a-ENSG00000163513 | Lung adenocarcinoma \|\| id:ieu-a-984 | Weighted mode | 5 | 0.0687961 | 0.068983 | 0.3921245 |
