# Supplementary material for: Identification of potential biomarkers for lung adenocarcinoma: a study based on bioinformatics analysis combined with validation experiments
Source: Front Oncol. 2024 Sep 19;14:1425895. doi: 10.3389/fonc.2024.1425895 (PMC11446723; doi:10.3389/fonc.2024.1425895)
Supplement: Supplementary file 1 [file DataSheet1.zip › Data Sheet 2/supplementary table/supplementary Table5.docx]

Supplementary Table5 Sensitivity analysis of reverse Mendelian randomization

| id.exposure | id.outcome | outcome | exposure | egger_intercept | se | pval |
| --- | --- | --- | --- | --- | --- | --- |
| ieu-a-984 | eqtl-a-ENSG00000163513 | ENSG00000163513 \|\| id:eqtl-a-ENSG00000163513 | Lung adenocarcinoma \|\| id:ieu-a-984 | -0.031679319 | 0.0382208 | 0.4943579 |
