# Supplementary material for: Identification of potential biomarkers for lung adenocarcinoma: a study based on bioinformatics analysis combined with validation experiments
Source: Front Oncol. 2024 Sep 19;14:1425895. doi: 10.3389/fonc.2024.1425895 (PMC11446723; doi:10.3389/fonc.2024.1425895)
Supplement: Supplementary file 1 [file Supplementary Fig.1(2).pdf › Data Sheet 2/Supplementary Fig.1(2).pdf]

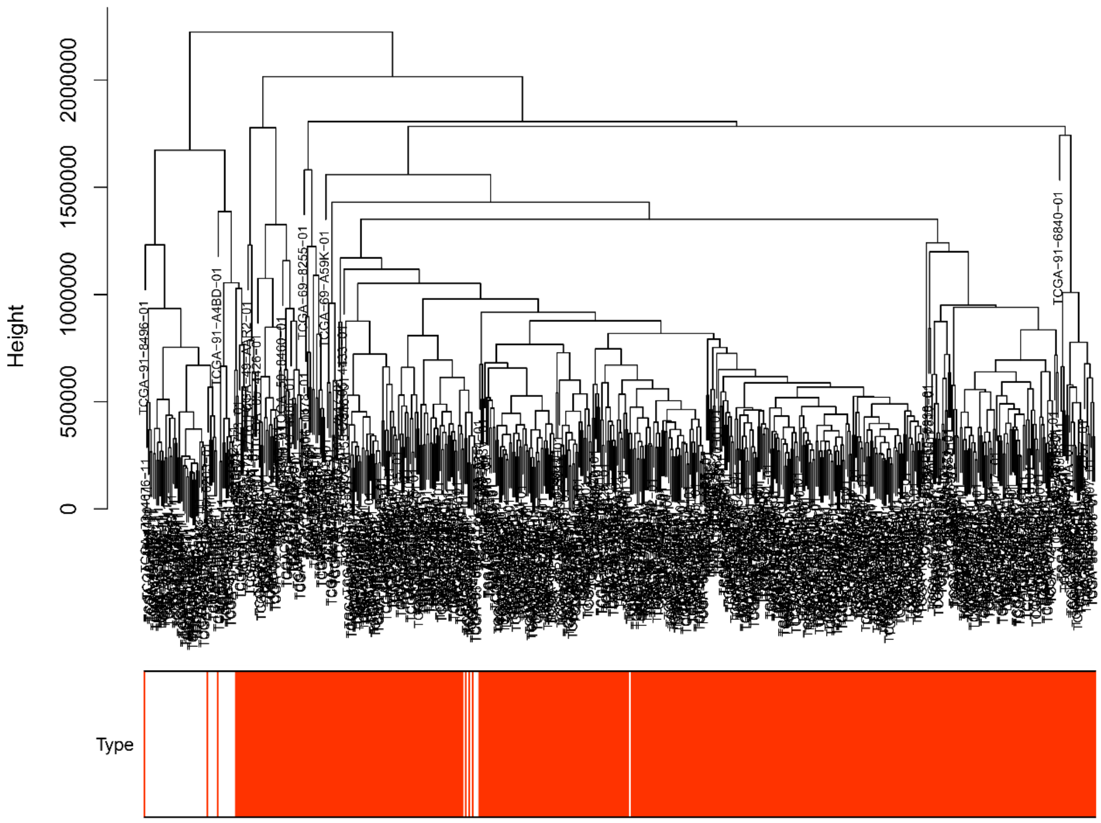

Supplementary Fig.1 Sample dendrogram and trait heatmap. Branches represent samples, and the vertical axis represents the height of hierarchical clustering. The red part in Type represents LUAD, and the white part represents the control.
